# MycoNews 2019: editorials, news, reports, awards, personalia, book news, and correspondence

**DOI:** 10.1186/s43008-019-0024-4

**Published:** 2019-12-30

**Authors:** David L. Hawksworth

**Affiliations:** 1 Comparative Plant and Fungal Biology, Royal Botanic Gardens, Kew, Surrey, TW9 3DS UK; 2 Department of Life Sciences, The Natural History Museum, Cromwell Road, London, SW7 5BD UK; 3 Jilin Agricultural University, Changchun, 130118 Jilin Province China

**Keywords:** Book reviews, Fungi, Meeting reports, Nomenclature, Obituaries, Taxonomy, Tributes

## Abstract

This first instalment of MycoNews includes: an Editorial “Do we need more governance in taxonomy?”; reports of mycological meetings in Poland (18th Congress of European Mycologists), Iran (4th Iranian Mycological Congress) and Chile (1st Chilean Meeting of Mycology (I Encuentro Chileno de Micología); an award to Lynne Boddy; birthday greetings to Gro Gulden, Marja Härkönen, Gregoire Hennebert, Hannes Hertel, and Junta Sugiyama; tributes to the passing of Francisco Calogne, Stanley J. Hughes, and Jos Wessels; news of four mycological books and one on-line work published in 2019; and a special tribute to Stanley Hughes by Kris Pirozynski.

## INTRODUCTION

*IMA Fungus* is the official journal of the International Mycological Association (IMA). Since it was launched at the 9th International Mycological Congress (IMC9) in Edinburgh in 2010, each issue has carried not only original research papers, but news and material on diverse topics of interest or concern to mycologists worldwide: editorials which may be controversial, news relating particularly to the work of the IMA, reports of international mycological meetings, awards and honours received by mycologists, birthday tributes to well-known and respected mycologists, obituaries, news of recently published mycological books, and correspondence which may be on any mycological issue. This tradition is now being continued as *MycoNews* by arrangement with the journal’s new publisher. *IMA Fungus* will also continue to include material of bibliographical, historical or ethnomycological material from time to time in the category *MycoLens,* combined reports on newly sequenced fungal genomes in a twice-yearly *Fungal Genomes* article, and material relevant to the journal’s mandated unique role in fungal nomenclature under the category *Nomenclature.*

*MycoNews* is compiled by myself as Editor-in-Chief, and to whom material for consideration for inclusion should be sent directly by e-mail, as should any that might be suitable for the *MycoLens* and *Nomenclature* categories, or items of correspondence. Reports of new fungal genome sequences should be sent for assessment of suitability to Senior Editor Brenda J. Wingfield, and books for possible coverage in the book news section to me at Milford House, 10 The Mead, Ashtead, Surrey KT21 2LZ, UK. All unsigned material in *MycoNews* can be attributed directly to myself.

## EDITORIAL Do we need more governance in taxonomy?

(Fig. 1)

The issue of governance in taxonomy has sparked considerable controversy following a plea in the correspondence column of *Nature* in 2017 by researchers primarily concerned with the conservation of mammals (Garnett and Christidis 2017). They identify a need for agreement on species concepts against a background of species being subdivided or united as a consequence of molecular evidence but without any consistency in approaches and leading to changes in conservation status. In order to deal with this situation, they suggested that the International Union of Biological Sciences (IUBS), of which the International Mycological Association (IMA) is a Scientific Member, establish an overall Commission on Taxonomy with a series of Subcommittees concerned with different groups of organisms which would provide standardized species lists. This resulted in a flurry of responses to *Nature,* of which six were published together just four weeks later; stances ranged from pointing out the current arrangements over nomenclatural rules (Funk et al. 2017), and heated objections to any constraint on scientific autonomy (Lambertz 2017), to support for a new Commission complementing existing nomenclaturally orientated bodies (Buckeridge 2017). The IUBS responded by including a workshop on “Governance of Biological Nomenclature” on 31 August 2019 during its centenary 33rd General Assembly in Oslo (20 July–2 August 2019); this was organized by the International Commission on Bionomenclature (ICB), and included presentations on both the concerns and the situation in various groups of organisms (including fungi). I understand that the matter is now to be pursued as an IUBS programme for the next 3 years, but further details on that are awaited and the IMA can expect to be involved.

**Fig. 1:**
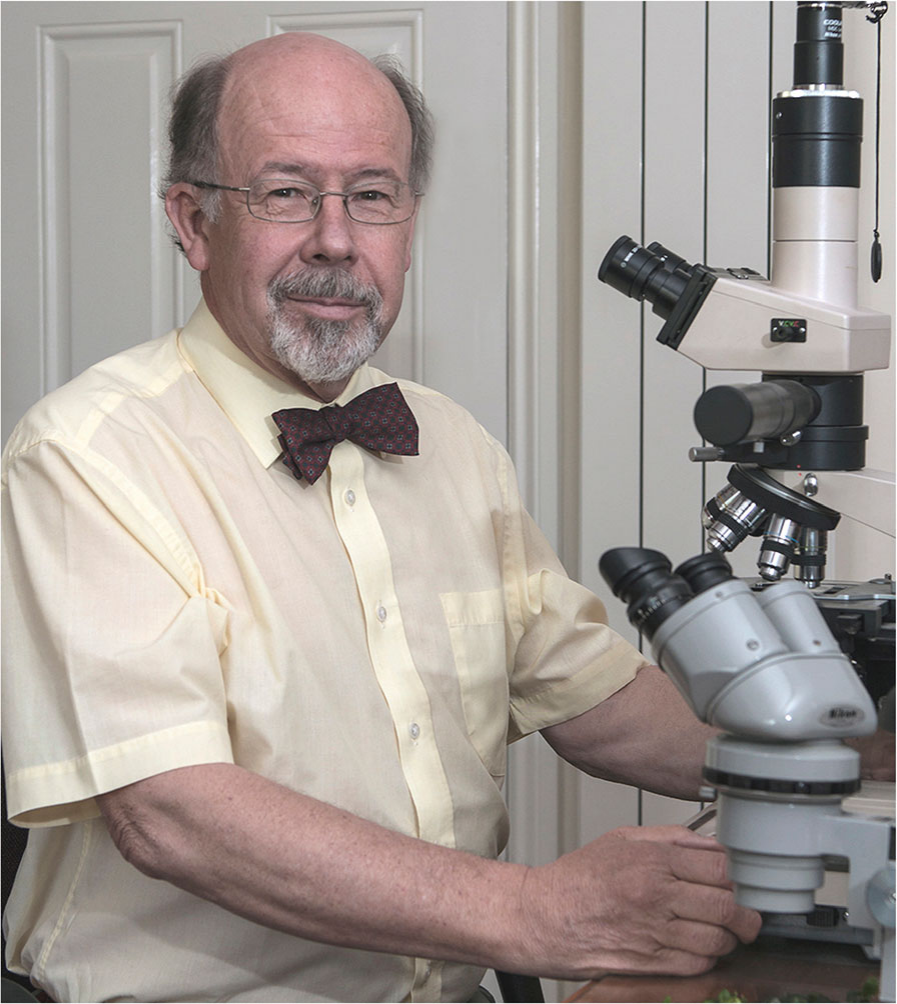
David Hawksworth

The proposal of having “standard lists” prescribing which names are to be used is, as with so many “good” ideas, not new. The first major list of which I am aware is that produced on behalf of the American Joint Committee on Horticultural Nomenclature; this started preparation in 1915 and published the first edition in 1932 and the names were to be adopted for not less than 5 years (Olmsted et al. 1932). At last we have in mycology the authority to produce lists of “protected names”, whether they are known to be threatened by other names or not, something first mooted in 1959 by the Danish mycologist Morten Lange (Gilmour 1959) but which was only fully realized in 2017 (Hawksworth et al. 2017). Unlike standard lists, protected lists do not, however, recommend any particular taxonomy but only rule on what names should be retained if they are threatened by any competing names.

Users of scientific names understandably become frustrated by repeated changes, especially when they are not specialists and do not understand the reasons for a particular change. This is a matter not just of the names of genera and species, but also of ranks right through from family to phylum. “Fiddling with names” and “ever changing the classification” have been perceived as failures in systematics which have contributed to the decline in its support (Bisby and Hawksworth 1991). In the case of fungi, this has become a huge irritation in recent years with the rapid advances being made through molecular systematics, and amongst field mycologists in particular (Jordan 2016). This is especially so as changes proposed by one research group may not be considered appropriate by another and conflicting classifications appear. Would the production of standard lists of fungal names to be adhered to for a fixed period be a partial solution to this problem?

Ainsworth (1966) aimed to do this for the rank of order and above through a “general purpose classification” designed to be used in editions of the *Dictionary of the Fungi* which was widely used as a standard reference framework. It is, however, vital not to constrain the proposing of new hypotheses to be debated and tested by the community, and an answer could be to distinguish between “generalist” classifications commended for widespread use and “specialist” classifications intended for consideration and debate by the scientific community (Hawksworth 1985). In the case of genera and higher ranks of ascomycetes, it was the vision of the annual series “Outline of the Ascomycetes” that started publication in 1982 to publish notes on proposed changes, by the compilers or others, and then incorporate those in the next annual Outline (Hawksworth and Eriksson 1994); the series continues today (Wijayawardene et al. 2018).

It was suggested in the Discussion section of the 1994 paper, that this initiative might be overseen by a body such as the International Commission on the Taxonomy of Fungi (ICTF). That was a considerable expectation at the time, but in recent years the situation has dramatically changed. In order to address changes in various groups of ascomycetes arising from the end of the separate naming of different morphs of the same species in 2011, and recommend which generic names were to be used, the ICTF established 19 working groups (Miller 2015). Many of these have already reported, and their conclusions will form the basis of lists of protected names. While the work of these groups has essentially been nomenclatural, focussed on competing generic names, what they have demonstrated is the feasibility of specialists around the world coming together to reach a consensus on what should be done.

In line with the moves being discussed under the new IUBS programme, it could be appropriate for the ICTF to now start to consider how it might best contribute to governing changes in classification at all levels. This might be achieved by extending the mission of its current working groups, or establishing new groups, charged with collaborating to produce an overall “generalist” classification across all groups of fungi, initially dealing with genera and higher ranks, perhaps with a view to this being updated every 5 years. In the absence of an edition of *Ainsworth & Bisby’s Dictionary of the Fungi* since the 10th (Kirk et al. 2008), the treatments in the 13th edition of the *Syllabus of Plant Families* (Jaklitsch et al. 2016; Begerow et al. 2018) form a valuable starting point for scrutiny by any newly mandated ICTF working groups. The issue of the ICTF potentially having a key role in the governance of fungal taxonomy could perhaps become a key issue for debate at the 12th International Mycological Congress (IMC12) in Amsterdam in 2022.

Mycologists have been ahead of both botanists and zoologists in establishing registration systems for newly published names, and now also the ability to safeguard names against all-comers through protected lists. Why should we not then also lead the way in commending names and classifications for general use?

## REPORTS 18th Congress of European Mycologists: Fungi in nature and culture

(Figs. 2, 3, 4)

The 18th Congress of European Mycologists (CEM 18) was held from 16 to 21 September 2019 in Warsaw and Białowieża, Poland. The event was organized by the Polish Mycological Society and European Mycological Association (EMA) with the support of several Polish universities and the Polish Academy of Sciences.

**Fig. 2:**
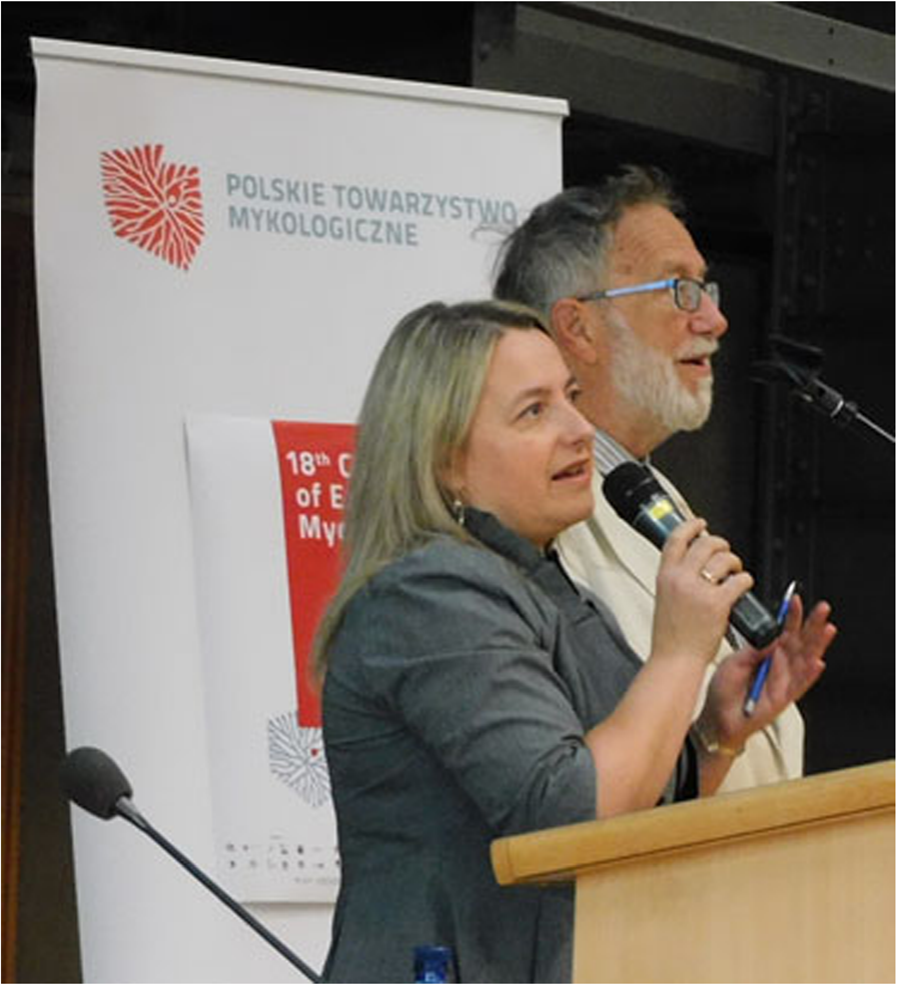
David Minter (President, European Mycological Association) and Magdalena Frąc (President, Polish Mycological Society) at the opening of the Congress

**Fig. 3:**
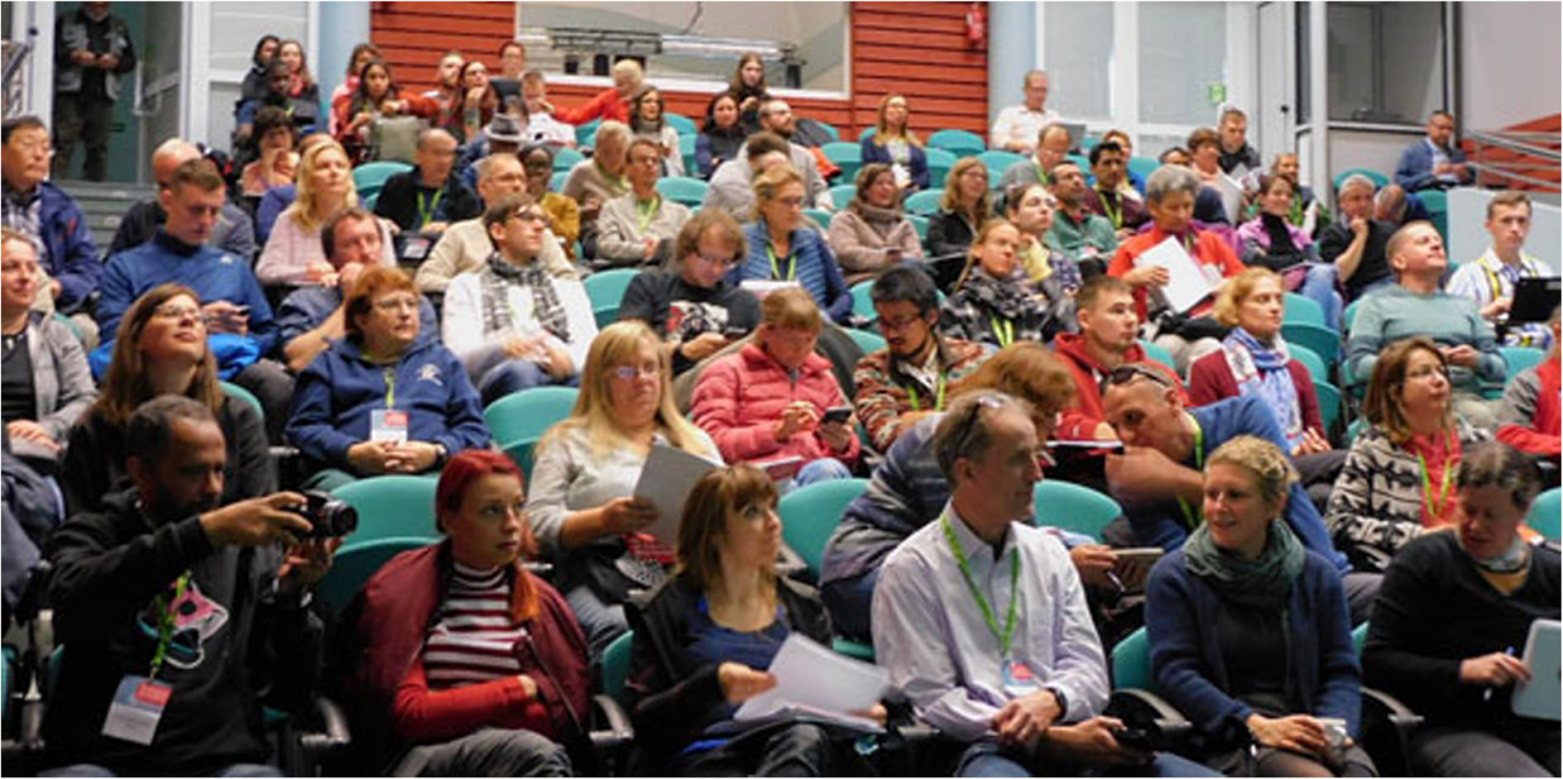
Some participants of the 18th Congress of European Mycologists in Białowieża

**Fig. 4:**
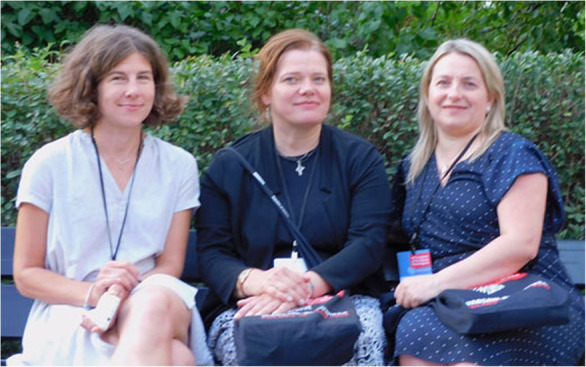
The CEM local organizing committee: Julia Pawłowska (*left*), Małgorzata Ruszkiewicz-Michalska (*centre*), and Magdalena Frąc (*right*)

The first 3 days were in the old campus of the University of Warsaw and devoted to fungal genetics, genomics, systematics, biotechnology and interactions, and the next 3 days in the Białowieża National Park where the topics centred on conservation and ecology. Participants were also able to visit the Strict Protected Area of Białowieża National Park and the Bison Reserve; this forest is generally recognized as the most ancient now present in central Europe. The welcome Reception was held in the Botanical Garden of the University of Warsaw, where the memory of the distinguished Polish mycologist Alina Skirgiełło (https://pbsociety.org.pl/journals/index.php/am/article/view/am.2008.014/2324 was honoured.

The Congress attracted 273 participants from 52 countries, and keynote lectures were presented by: Annegret Kohler (France), David Hawksworth (UK), Geoffrey Gadd (UK), Duur K. Aanen (The Netherlands), Dominik Begerow (Germany), Marc-André Selosse (France), Bogdan Jaroszewicz (Poland), and Lynne Boddy (UK). There were 77 other lectures and 176 posters, with sessions on the themes: From genome to function, Taxonomy and systematics, Fungi in biotechnology, Fungal interactions, Medical Mycology, Fungal diversity, Fungi in primeval forests and other natural habitats, Hypogeous mycorrhizal fungi, Fungal Conservation, and Data. Additionally, workshops were organised on Global fungal red-listing and the Biology of polypores. On the final Saturday morning, there was an open European Committee for Conservation of Fungi (ECCF) discussion forum on “Fungal conservation across borders in Europe (and beyond) for the next 4 years - what role for the ECCF?” Mushroom hunting was organized on the Friday morning and an iNaturalist.org BioBlitz project was created as a follow-up of a Global Biodiversity Information Facility (GBIF) presentation that had stressed the importance of sharing data on fungal occurrence; 750 observations were made and 217 species of fungi recorded in the Białowieża Forest.

In keeping with the motto of the meeting, several exhibitions and events were organized, including “Fungi – master sculptures of nature”(photographs), “Dystopia”(a spatial composition by Hélène Soulier and Ewa Rudnicka), “Anomalium” (a graphics exhibition of Agnieszka Zdziabek), “Broken links”(a performance by Maria Subczyńska), and two mushroom exhibitions in Warsaw. An evening public lecture “Mycology: a recent weapon in the forensic armoury” by Patricia Wiltshire was given in the Copernicus Science Centre.

At a general meeting of the European Mycological Association (EMA) on 17 September, a new board was elected: Izabela Kałucka (President, Poland), Mitko Karadelev (Vice-President, Macedonia), Katerina Rusevska (Secretary, Macedonia), Eske De Crop (Treasurer, Belgium), Vijai Kumar Gupta (Membership Secretary, Estonia), Tatiana Semenova-Nelsen (Meetings Secretary, USA), Paulo de Oliveira (Webmaster, Portugal) and Susana C. Gonçalves (Conservation Officer and ECCF Chair, Portugal). The outgoing President, David Minter (UK), was thanked for all he had done for the EMA during his tenure.

During the Closing Ceremony, it was agreed that the 19th Congress of European Mycologists would be organized in Italy in 2023 in a venue to be announced later.

**Julia Pawłowska**

(jzpawlowska@gmail.com)

## 4th Iranian Mycological Congress

(Fig. 5)

The 4th Iranian Mycological Congress was held on 26–28 August 2019 at the Sari Agricultural Sciences and Natural Resources University (SANRU), Sari, Mazandaran, by the Iranian Mycological Society (IMS) in collaboration with SANRU. Iranian mycological congresses are planned to occur every 2 years; the first was at the University of Guilan (Rasht, Guilan) in 2013, the second at the University of Tehran (Karaj, Alborz) in 2015, and the third at the University of Kurdistan (Sanandaj, Kurdistan) in 2017.

**Fig. 5:**
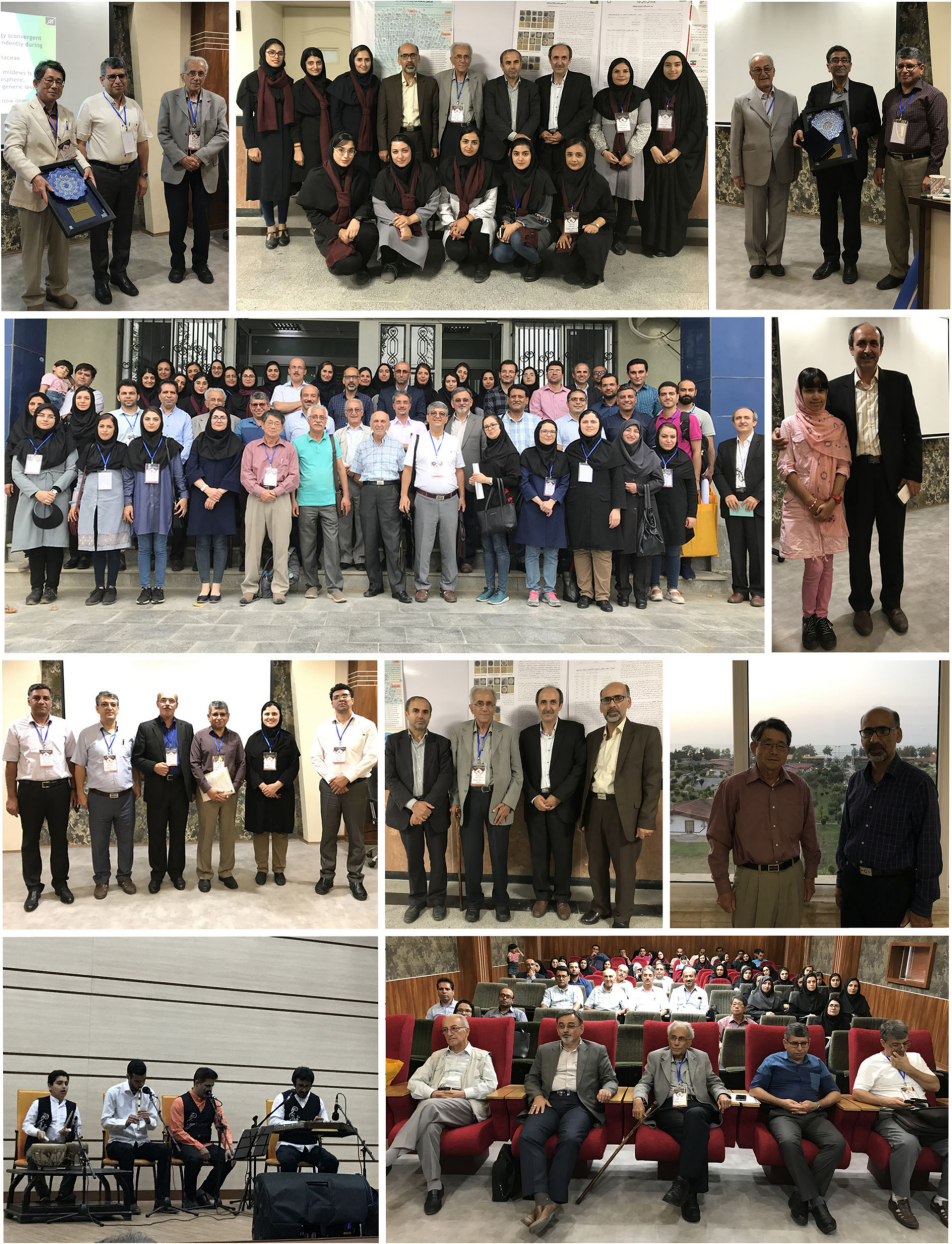
Scenes from 4th Iranian Mycological Congress

The congress consisted of diverse programmes, and the highlights included: Five keynote and 32 plenary lectures. The keynote speakers were Susumu Takamatsu (Japan), Mounes Bakhshi, Mohammad Sohrabi, Mohammad Reza Asef, and Adel Pordel; 103 poster presentations; and special symposia on the challenges and approaches for improving mycology teaching in Iran (during which the production of mycology textbook and a handbook for laboratory methods of mycology in Persian were proposed), and challenges and future prospects for molecular identification. Congress participants ranged from distinguished but now retired but still active mycologists (Djafar Ershad, Ghorbanali Hedjaroud, Zia Banihashemi, and Abdolghayoum Ebrahimi) to a 13 year-old girl (Hasti Tajik-Ghanbari). Other activities included a tour to the shore of the Caspian Sea, a dinner featuring Mazandaran folk music, also organized for the congress delegates, and post-congress workshops on molecular identification and the epiphytic lichens of the Hyrcanian forests. At the closing ceremony, Susumu Takamatsu was presented with an award of Excellence in recognition and appreciation of the quality, originality and quantity of his published research, especially on powdery mildews, and services to mycology; he was also elected as an honorary member of the Society.

The 10th General Assembly of IMS was held during the Congress, when a new board was elected: Mohammad Javan-Nikkhah (President), Bahram Sharif-Nabi (Vice-president), Mahdi Arzanlou, Mounes Bakhshi (Secretary), and Amirreza Amirmijani (Treasurer). The third Dr. Hedjaroud’s Award (see *IMA Fungus* 6(2): (47)–(48), 2016) was presented to Rasoul Zare in recogition of his valuable services to the development of the field of mycology in Iran.

As members of the Organizing Committee, we are very happy and delighted that the programme was so successful and the events so memorable.

**Mounes Bakhshi, Rasoul Zare, and Akbar Khodaparast**

(mounesbakhshi@gmail.com)

## 1st Chilean Meeting of Mycology (I Encuentro Chileno de Micología)

(Figs. 6, 7, 8)

The first ever Chilean Meeting of Mycology was held on 5-6 September of 2019 at the María Ghilardi Auditorium of the Science Faculty of the University of Chile in Ñuñoa Metropolitan Region. The event attracted more than 200 participants, and comprised five symposia, two invited talks, 20 oral presentations, and 31 posters. Most attendants were from Chile, but some researchers from Brazil and Mexico also joined the occasion. The meeting had three themes: Fungal biodiversity, ecology, biogeography and conservation; Mycorrhizas: from fundamental to applications; Fungal uses and applications: Ethnomycology, biotechnology, chemistry and medical mycology; and Taxonomy of Fungi, lichens and myxomycetes. A pre-meeting course led by Patricia Silva-Flores gathered 17 people together to share knowledge on mycorrhizas of the sclerophyllous forest of Central Chile.

**Fig. 6:**
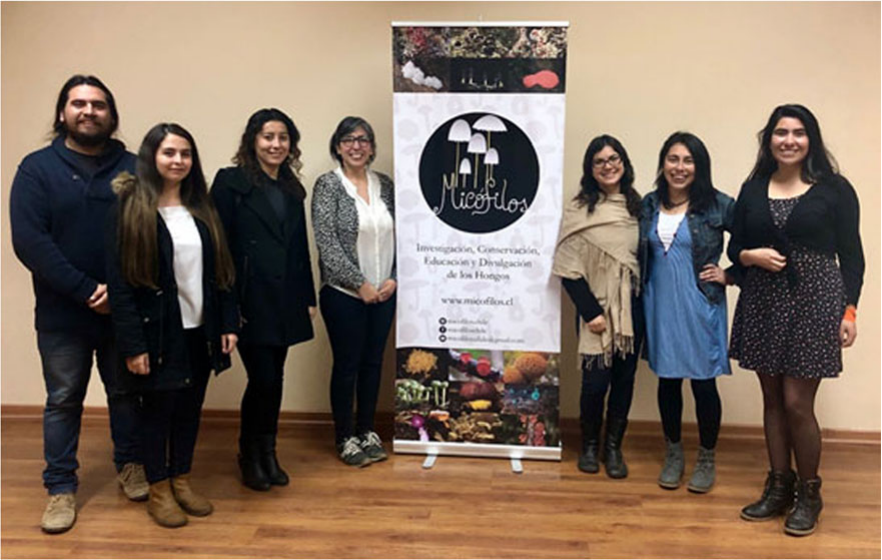
The organizing team from Micófilos (*left to right*): Christian Valdés, Sandra Troncoso, Viviana Salazar-Vidal, Patricia Silva-Flores, María José Dibán, Dinelly Soto, and Francisca Narváez; Valentina Cáceres is missing

**Fig. 7:**
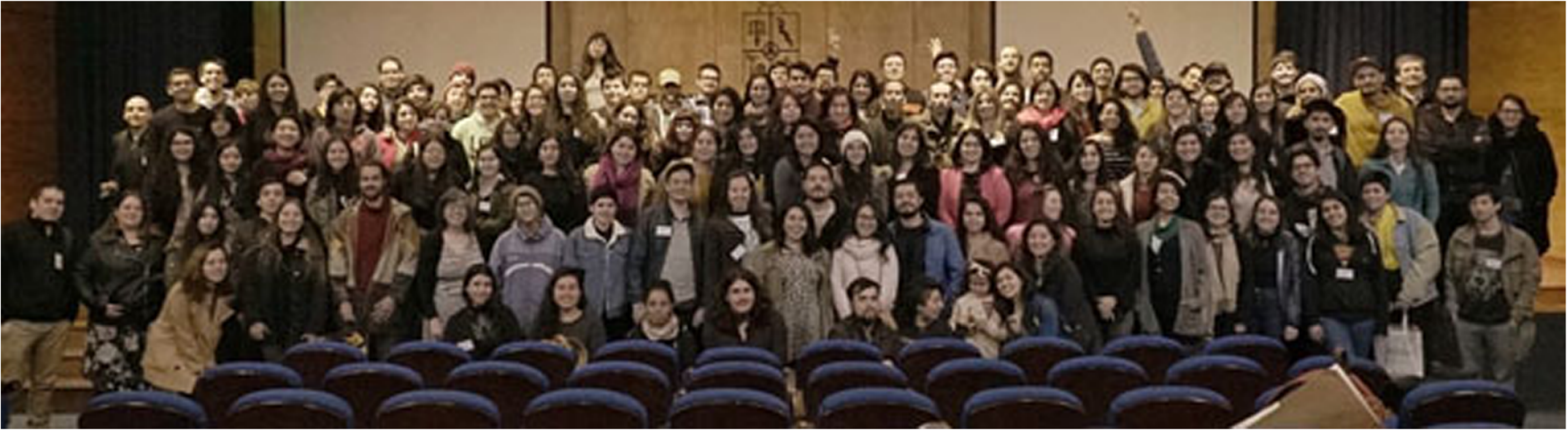
Participants of the 1st Chilean Meeting of Mycology

**Fig. 8:**
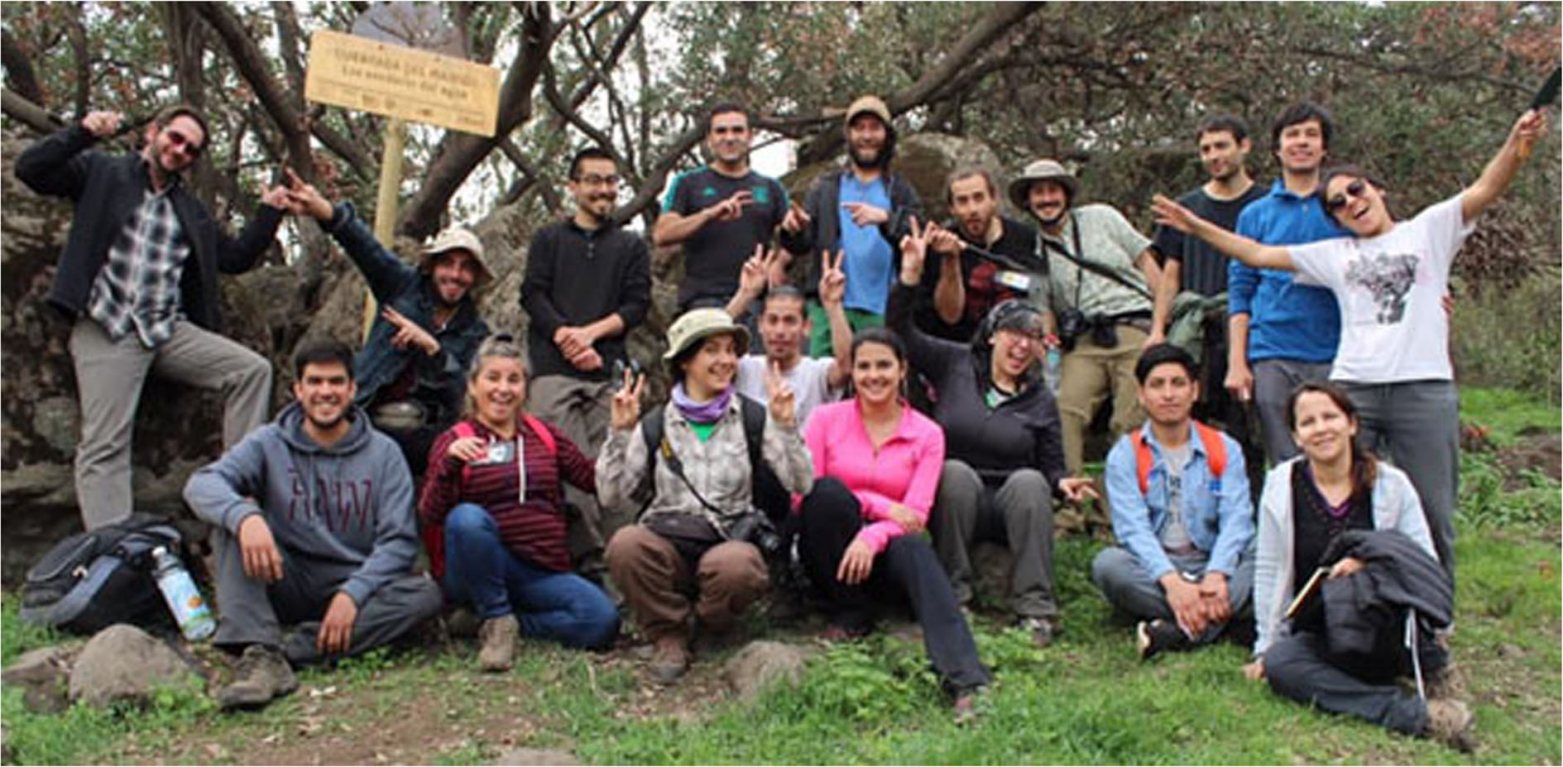
Participants in the pre-meeting course “Introduction to mycorrhizas of the Chilean sclerophyllous forest”

The initiative to organize this event came from Viviana Salazar-Vidal and Sandra Troncoso, both biologists and Master students from the University of Concepcion; they talked and dreamed about a meeting to gather Chilean mycologists together to share knowledge, create collaborative networks, and outreach to the community. The torch was taken up by Micófilos (https://www.micofilos.cl), a non-governmental organization (NGO) dedicated to research, conservation, environmental education, and scientific outreach on fungi and fungal-like organisms in Chile. Micófilos was also established by Viviana Salazar and formalized in 2018 with the support of several professional mycologists. Valuable support of several people and entities helped realize this initiative, not least Waldo Lazo (author of the first Chilean Mycological Atlas) and several Chilean universities and other organizations.

We found that there were at least 57 professionals distributed in several centres in different regions of Chile researching mycological topics and training students, demonstrating a promising future for mycology in the country. Awards were given for the best oral and poster presentations, and Waldo Lazo and Viviana Salazar were recognized for their contributions to Chilean mycology. The meeting ended with a mushroom tasting, where chef Marco Maldonado prepared multiple dishes based mainly on *Morchella*, *Suillus,* and *Pleorotus* species.

We consider this event a milestone in Chilean mycology, since the Chilean mycologists never before had the chance of meeting and presenting their research in a local forum dedicated exclusively to fungi. It was an enriching instance of learning and sharing experiences not only with professional researches, but also amateur mycologists and the local community as the event was open to anyone willing to learn about fungi., strengthening the bridge of researchers with the community through scientific outreach. We plan to organize a second meeting in 2021, where we hope to also welcome participants from outside Chile.

**Patricia Silva-Flores, Viviana Salazar-Vidal, María José Dibán, Sandra Troncoso, Francisca Narváez, Dinelly Soto, Valentina Cáceres, and Christián Valdés**

(psilva@ucm.cl)

## AWARDS Lynne Boddy MBE

(Fig. 9)

We are pleased to record that Lynne, based at what is now the University of Cardiff since 1983, and as Professor of Mycology since 1996, has been appointed a Member of the British Empire (MBE) for “Services to Mycology and Science Outreach”. Recipients of these national honours, which are presented by Queen Elizabeth II or another senior member of the Royal Family, are selected after rigorous scrutiny by committees of the Cabinet Office and cover all kinds of exceptional personal achievements. The IMA is so pleased that her work has been recognized in such a public way.

**Fig. 9:**
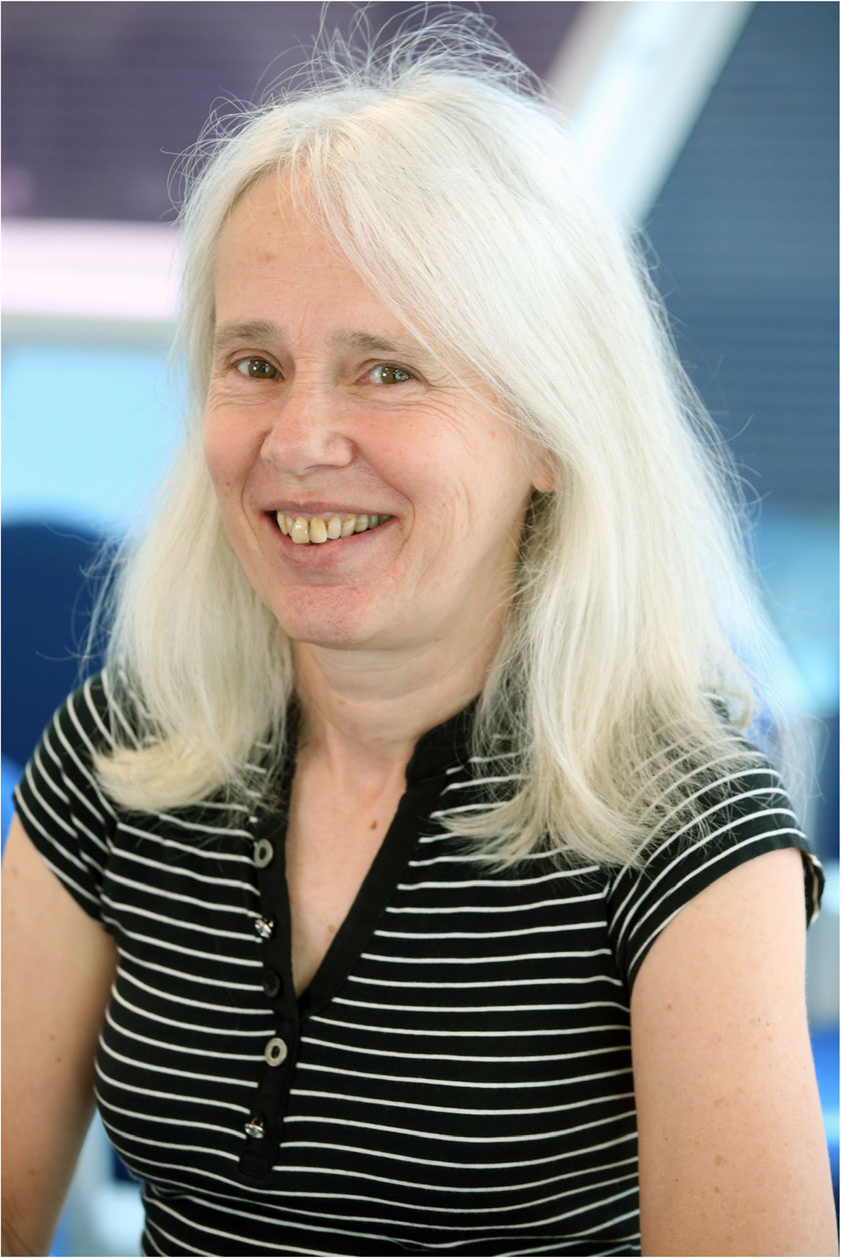
Lynne Boddy MBE

Lynne, who was President of the British Mycological Society for 2009–10, has focussed her career on fungal ecology, particularly the decomposition of wood and the interactions of fungi involved in wood decay, more recently also on effects of climate change, and is the founding Editor-in-Chief of *Fungal Ecology* (launched in 2008). An enthusiastic and much sought-after speaker, Lynne has done much to improve the public understanding of fungi not only through her lecturing and writings, but through radio and television interviews and spectacularly the *From Another Kingdom: amazing world of Fungi* exhibition and lavishly illustrated book launched at IMC9 in Edinburgh in 2010. She is also renowned for her encouragement and training of graduate students which we trust will continue for many years to come.

You can listen to Lynne speaking about her work in the *Oral History of Mycology* interviews made last year at IMC11 in Puerto Rico (https://www.youtube.com/watch?v=pXWEux2DPGA).

## BIRTHDAY GREETINGS Grégoire Laurent Hennebert

(Fig. 10)

It is difficult to believe that the ever-sprightly Grégoire turned 90 on 20 June this year! Born in Mons, Belgium. He studied at the University of Louvain, receiving his doctorate in 1956 for studies on *Botrytis*, and soon after went to Ottawa for 2 years where he came under the guidance of Stanley (“Stan”) J. Hughes, James W. Groves, and Luella K. Weresub. He returned to Louvain in 1962, becoming a researcher, lecturer, and later professor, organizing the fungal collections held there into the Mycothèque de l’Université Catholique de Louvain (MUCL) in 1972 – and then having to oversee its move to Louvain-la-Neuve in 1975. MUCL grew into one of the most important collections of fungal cultures in Europe, now with over 30,000 strains. Grégoire spent two weeks each year at the then Centraalbureau voor Schimmelcultures in Baarn, The Netherlands, from 1964 to 75, also visiting Cornell University in the USA where he developed a close collaboration with Richard (“Dick”) P. Korf. That collaboration resulted in the launching of the still flourishing journal *Mycotaxon* in 1974, for which he prepared book reviews through to 1995.

**Fig. 10:**
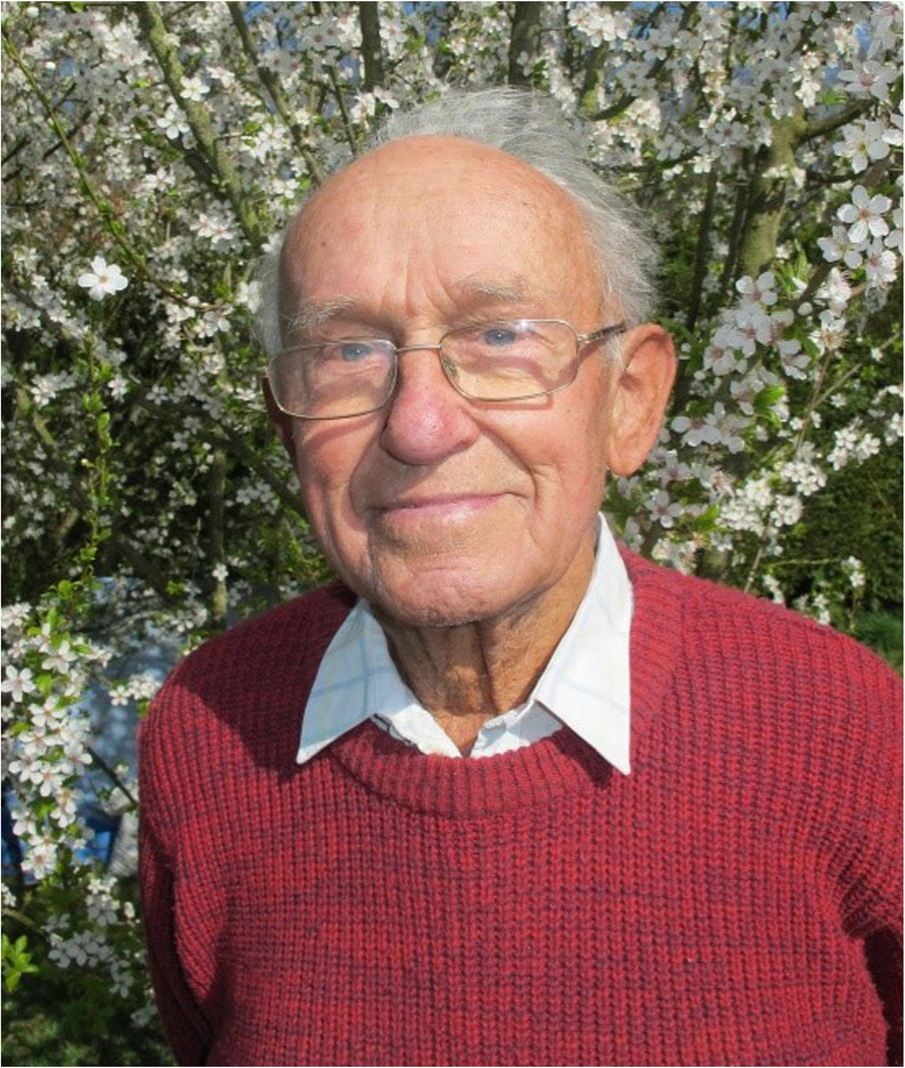
Gregoire Hennebert

His mycological interests are extraordinarily wide, embracing not only systematics of his beloved hyphomycetes, but also the cultivation of edible fungi, yeasts, and biodegradation. Always an original thinker and interested in nomenclature and terminology, he served on what was then the Special Committee for Nomenclature of Fungi and Lichens for many years, and with Luella Weresub was responsible for introducing the terms anamorph/teleomorph/holomorph into mainstream mycology in 1977 – and discussed in more detail later (Hennebert 1987). With an interest in Africa from his student days, Grégoire has done much to encourage the development of mycology in the French-speaking countries in the continent, especially through guiding fungal research in the University of Burundi. He also edited and published papers presented at the first regional conference on mycology in Africa, which took place in Mauritius in 1990, and later published a first *Directory of African Mycologists* (Buyck and Hennebert 1994).

Active on numerous committees and seemingly always there at key mycological workshops and conferences, with an often wry smile, he continues to be productive. Indeed, I understand he currently has two papers in press and another on *Chromelosporium* in preparation! The IMA wishes him well on the occasion of this very special birthday, and trusts he will continue to enjoy life with the fungi he loves, his mycological colleagues, and his family.

## Gro Gulden

(Fig. 11)

Gro Gulden celebrated her 80th birthday this year. She was educated as a mycologist at the University of Oslo and subsequently became curator of fungi at the Norwegian Natural History Museum. Her interest is the agarics, and she specialized in the difficult genus *Galerina* from northern Europe. From 1980 onwards, she treated the genus in alpine Norway, Svalbard, Iceland, Faroe Islands, Greenland, and the Nordic countries, terminating with a summary of 30 *Galerina*s in cold climates in 2010, following DNA-studies in this polyphyletic genus in 2005. Other cold climate studies include an agaric funga for Svalbard and, in collaboration with K. Mohn Jenssen, a series of booklets with loose plates that could be rearranged as more volumes appeared. She illustrated many arctic and alpine fungi for the first time, which have contributed much to our knowledge of these difficult to access species.

**Fig. 11:**
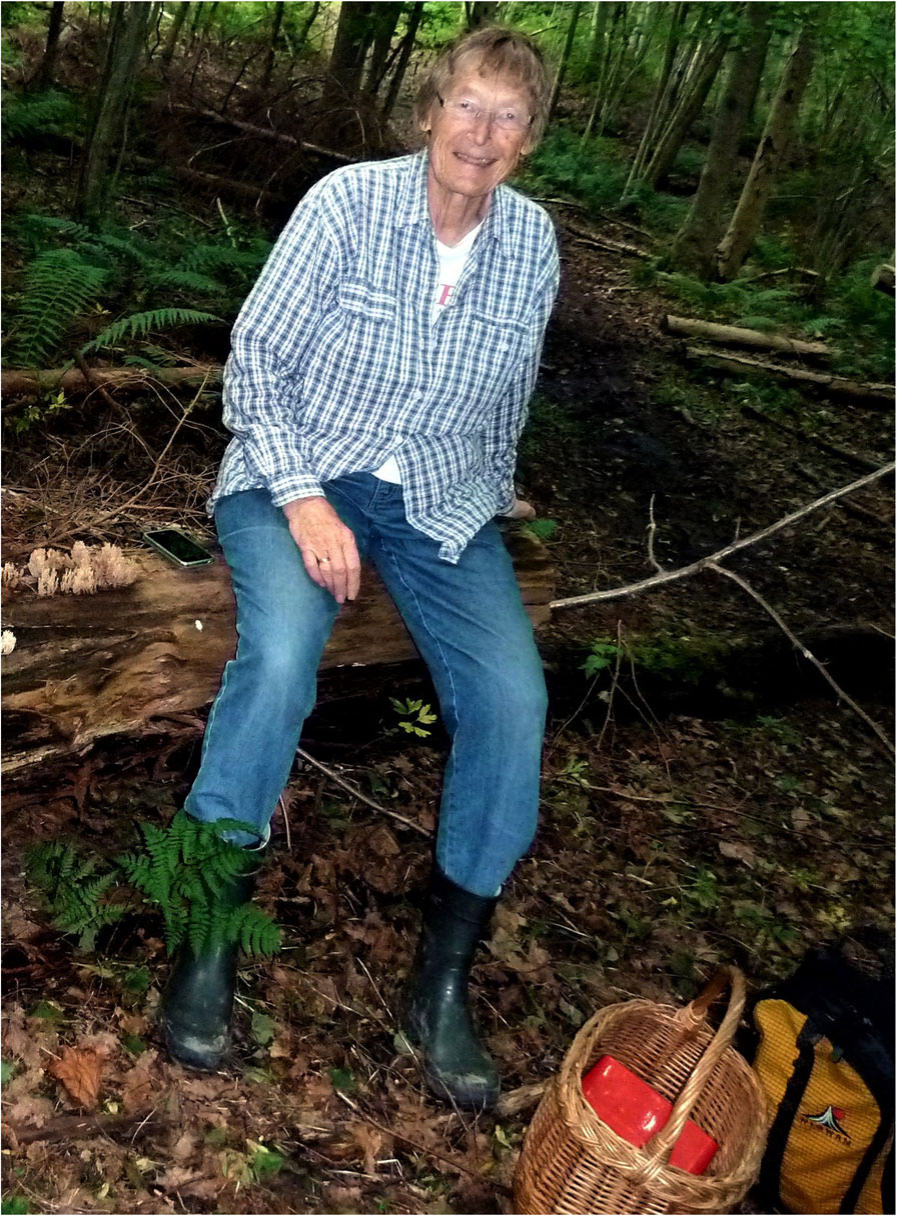
Gro Gulden. Photo: Inger Kristoffersen

In the 1980s Gro participated in a study of the hydnoid mycorrhizal *Thelephorales*, prompted by alarming reports from The Netherlands of a marked decline in these fungi. These studies continue and now focus on the enigmatic resupinate species in the group.

Internationally, Gro has participated in the International Symposium on Arctic and Alpine Mycology (ISAM) series from the first in Alaska in 1980, and in 2005 she arranged ISAM 7 in Norway. She is a pioneer in the study of cold climate fungi with many contributions and actively promoting the subject.

Gro is the grand old Lady of Norwegian mycology, always helping, initiating, promoting and supporting mycology and connecting people with similar interests. We send her our congratulations on the occasion of this special birthday, and wish her a continuing fruitful career in the service of mycology!

**Henning Knudsen**

(henningk@snm.ku.dk)

## Marja Härkönen

(Fig. 12)

Marja Härkönen celebrated her 80th birthday on 17 October 2019. Marja was born in Tampere, Finland, and obtained an MSc from the University of Turku in 1968. She worked as a biology and geography teacher before being appointed as an assistant in the University of Helsinki in 1974, and defended her PhD thesis *Ecological, taxonomical and chorological studies on Finnish Myxomycetes* in 1979. Before retirement in 2003, she held several teaching positions in the university including Associate Professor, Acting Professor of Botany, and Acting Professor of Mycology, and many other positions of trust both at the University and in governmental organizations. After her first article on Finnish slime moulds in 1974, Marja published on myxomycetes from many parts of the world; her publication list includes 260 scientific and popular titles. *Limasienet* [The myxomycetes of Finland] (Härkönen and Sivonen 2011) was a landmark in Finnish myxomycology: an identification guide describing all 204 species known from the country at that time and providing new names for no less than 202 species! The beautiful book immediately generated new interest on slime moulds, and ten new species records were already added to the second edition in 2012. Together with Tuomo Niemelä and other colleagues Marja produced two mushroom guides for East Africa: *Tanzanian Mushrooms: edible harmful and other fungi* (Härkönen et al. 2003) and *Zambian Mushrooms and Mycology* (Härkönen et al. 2015) based on extensive interviews with local peoples. We understand she is currently working on another field guide on East African fungi, and wish her well in this and all her future endeavours.

**Fig. 12:**
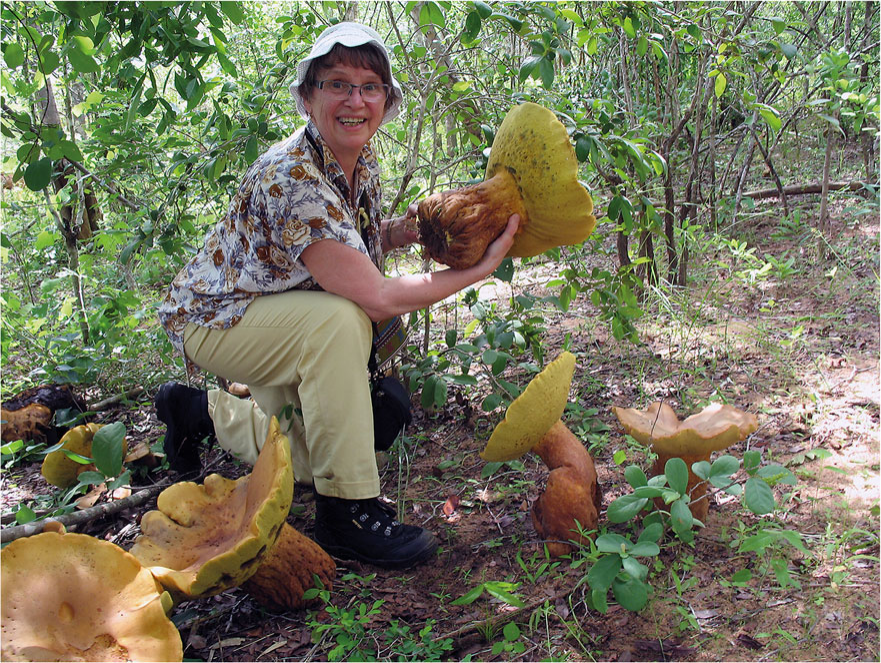
Marja Härkönen collecting *Phlebopus sudanicus* in Mozambique, 2011

**Jouko K Rikkinen**

(jouko.rikkinen@helsinki.fi)

## Hannes Hertel

(Fig. 13)

Hannes Hertel, an eminent lichenologist and long-standing curator at the Botanische Staatssammlung München (M), celebrated his 80th birthday on 3 February 2019. Born in Munich, as a young student he developed a profound interest in vascular plants and their associations. His mentor and supervisor Josef Poelt turned his interests towards lichens, and in 1967 he published his doctoral thesis on calciphilous species of *Lecidea*. In 1972 he became a professor at the Freie Universität Berlin, but moved back to Munich the following year to become curator at the State Herbarium, which he led as provisional director from 1985 to 1992. Hannes retired in 2004, but is still actively pursuing studies, now mostly on the history of lichenology. In 2008 the International Association for Lichenology (IAL) awarded him the Acharius Medal for his lifetime’s work as a lichenologist. Over the years, three genera and 16 subgeneric taxa of lichenized fungi have been named in his honour.

**Fig. 13:**
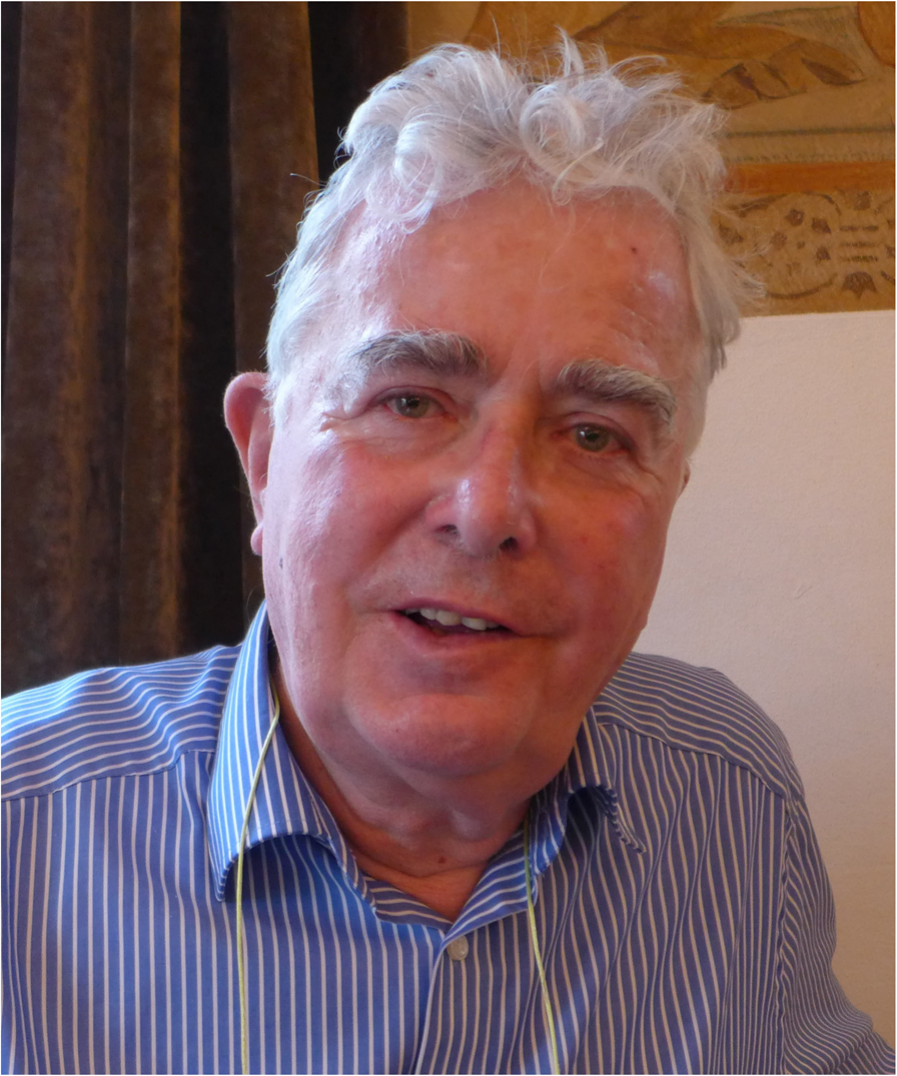
Hannes Hertel in his Munich home, 24 October 2019. Photo: Roman Türk

The huge form genus *Lecidea*, on which he specialized early on in his career, offered enough material for his own career of more than 40 years as a taxonomist as well as several master and PhD theses conducted under his supervision. His studies initiated a process that over decades resolved the systematic position of most species in this originally highly artificial taxonomic conglomerate. His most influential papers comprise revisions of European, Asian, Arctic, and Subantarctic species. His meticulous taxonomic studies were complemented by two exsiccate series (*Lichenes Alpium* and *Lecideaceae exsiccatae*) that still serve as reference standards for identification. His outstanding qualities as a teacher and mentor are reflected in four of his doctoral students pursuing academic careers as lichenologists or other mycologists.

Besides his merits as a worldwide recognized expert in lecideoid lichens, Hannes has always had a keen interest in making the rich lichen collections in Munich more accessible to the scientific community. His monumental indices of collectors and exsiccatae in M and his index to F. Arnold’s collecting localities in the Alps, recently complemented by an index to researchers on Austrian lichens, are extremely helpful tools (even in these times of searchable collection databases).

We send him our congratulations and wish all the best to a truly inspiring teacher, great scientist, and also a humble and most generous person!

**Christian Printzen**

(christian.printzen@senckenberg.de)

## Junta Sugiyama

(Fig. 14)

Junta Sugiyama, Emeritus Professor in the University of Tokyo, was born in Tokyo in 1939 and celebrated his 80th birthday on 25 January 2019. He graduated from Yokohama City University in 1962, where he attended a special mycology lecture by Yosio Kobayasi and was guided by Keisuke Tubaki and Masami Soneda at the Nagao Institute, graduating with a study on yeasts isolated from plant exudates. He went on to obtain a PhD from the University of Tokyo in 1969 for studies on the fungi in core samples from stratigraphic drillings in Japan. During this period he was involved in a microbiological survey of the Dry Valleys of Antarctica. After studying as a postdoc with “Stan” Hughes in Ottawa in 1970–1972 working on *Monodictys* and *Xylohypha*, he returned to take a post as a Senior Scientist in Mitsubishi Chemical Industries Ltd in Yokohama. In 1980 he moved back to the University of Tokyo, returning for a time to pleomorphic fungi, especially through sooty moulds (Sugiyama 1987), then being promoted to Professor in 1989, and Professor Emeritus on his retirement in 1999.

**Fig. 14:**
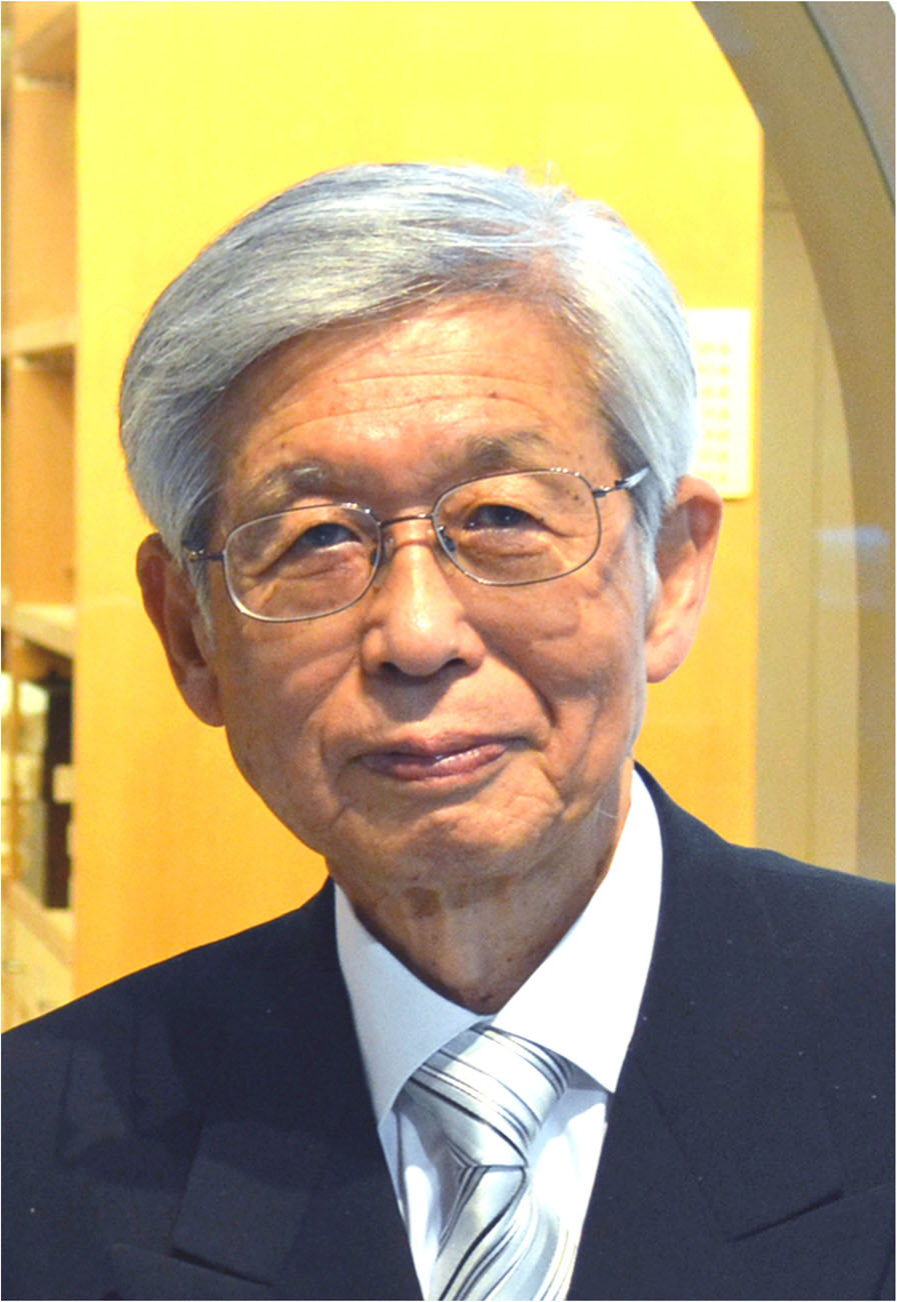
Junta Sugiyama, Minakata Kumagusu Archives, Tanabe City, Wakayama, Japan, May 2013. Photo: T. Kiyuna

He later became active in the National Collection of Industrial and Marine Bacteria (NCIMB) in Shizuoka and from 2016 has been a Visiting Researcher at the National Museum of Nature and Science in Tsukuba. He has received numerous honours and awards, and served the IMA on the Executive Committee (1994–1998) and as Vice-President (1998–2002), receiving the Fellow Medal in 2018. He was also active in the International Commission on the Taxonomy of Fungi (ICTF) from 1990 to 2002.

Junta is especially renowned for his pioneering innovative chemotaxonomic and molecular phylogenetic studies, not least on multiple losses of flagella in the chytridiomycetes-zygomycetes line, and the blending of phenotypic and genotypic characters in *Taphrina*, *Protomyces*, *Saitoella*, and *Mixia*. Active in the global Assembling the Fungal Tree of Life (AFTOL) project, most recently he has been involved in elucidating the cause of biodeterioration of 1300 year-old multicoloured mural paintings in Japan.

We thank him for his excellent leadership in mycology, and send him our hearty congratulations for 80th birthday as a great scientist, and hope for his continuous guidance to students of the mycological world.

**Takayuki Aoki**

(taoki@affrc.go.jp)

## IN MEMORIAM Francisco de Diego Calonge (1938–2019)

(Fig. 15)

Francisco “Paco” de Diego Calonge was born on 26 January 1938 at Chinchón (Madrid). He graduated in pharmacy from the Universidad Complutense de Madrid, and went on to submit a doctoral thesis in phytopathology. In 1965 he moved to the University of Bristol (UK) to carry out studies on the ultrastructural analysis of parasitic fungi, a field in which he made important contributions. Returning to Madrid in 1968, he joined the Real Jardin Botanico (MA; Consejo Superior de Investigaciones Científicas, CSIC) to which he devoted his professional life, rising to become a research professor in 1982 and Director of the whole garden from 1979 to 84. During his tenure, the Garden was restored and opened to the public in 1981.

**Fig. 15:**
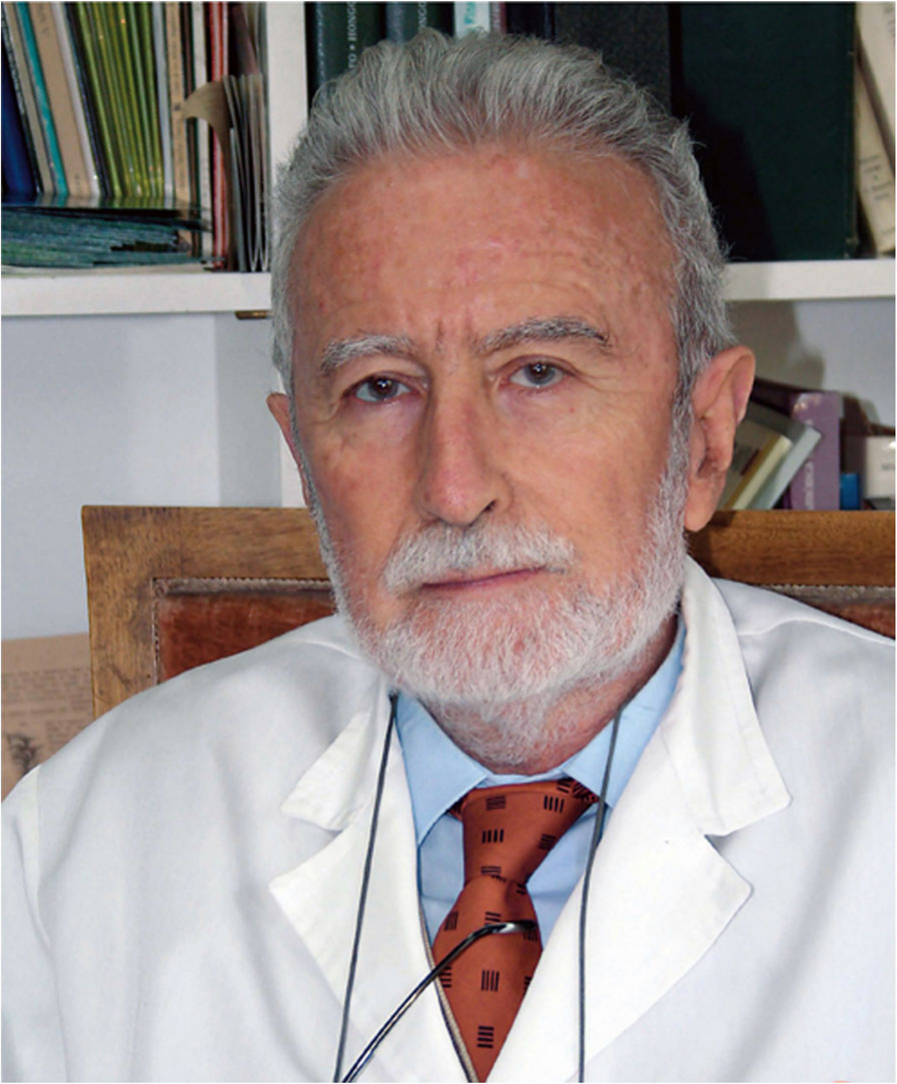
Franisco Calogne

When Paco rejoined the Spanish scientific system, he began floristic and taxonomic studies of macromycetes, especially gasteroid fungi – at first in Iberia, but then with international collaborators in the rest of Europe, and especially Madeira. He published numerous works on mushrooms from Bolivia, Brazil, Costa Rica, Ecuador, Guatemala, Mexico, and Venezuela; and further additions to those of Cameroon, India, and Tanzania.

He was a forerunner of scientific dissemination in Spain and was one of the greatest exponents in the field of mycology. He founded the Sociedad Micológica de Madrid, organizing annual mushroom exhibitions every fall from 1973, evening public identification sessions in the mushroom season demonstrating recent finds, the publication of a variety of field guides, and also numerous excursions. He is also renowned as a mentor and a supervisor of numerous doctoral theses. A warm and generous man, loved by all mycologists who came to know him, “Paco” died on 5 November 2019.

**María T. Telleria and María P. Martín**

(maripaz@rjb.csic.es)

## Stanley John Hughes (1918–2019)

(Fig. 16, 17)

It is with the utmost regret that we report the death of “Stan” in Ottawa on 7 November 2019 at the grand old-age of 101 years. Born in Llanelly, South Wales on 17 Sept 1918, “Stan” worked as an Assistant Mycologist at the then Commonwealth Mycological Institute (CMI) in Kew from 1945 to 52, where he was much influenced by its’ first mycologist, Edmund W. Mason. In 1949 he spent 3 months in Ghana (then the “Gold Coast”) becoming fascinating by the variety of unrecognized tropical microfungi. He published a staggering 16 of the Institute’s *Mycological Papers* during this time (and then a 17th in 1993). Stan then moved to the Central Experimental Farm (CEF) of Agriculture Canada in Ottawa, first distinguishing eight main types of conidiogenesis (Fig. 17), and then using that scheme devoting himself to putting the hyphomycete fungi into order; this involved studying the application of generic names by studying the classical mycological literature and locating and examining type material in the major European collections. He also established the numbering and record book cross-indexing system of the fungal collections in CEF that had become familiar with in Kew, enabling material to always be re-located when moved around in the collections and those known from different host plants accessed. After spending a full year based in Auckland, New Zealand, in 1962–63, the microfungi of that country became a particular interest and resulted in a a series of 36 papers in the *New Zealand Journal of Botany* running from 1964 to 2003. This also led led him undertake the daunting task of rationalizing our knowledge of sooty moulds, which involved re-assessments of the often several asexual morphs of single species; he thought these were “lovely” and his eyes would sparkle when he talked about them. His papers were always meticulously illustrated by both line drawings and photographs – though he eventually conceded that life was too short for stippling.

**Fig. 16:**
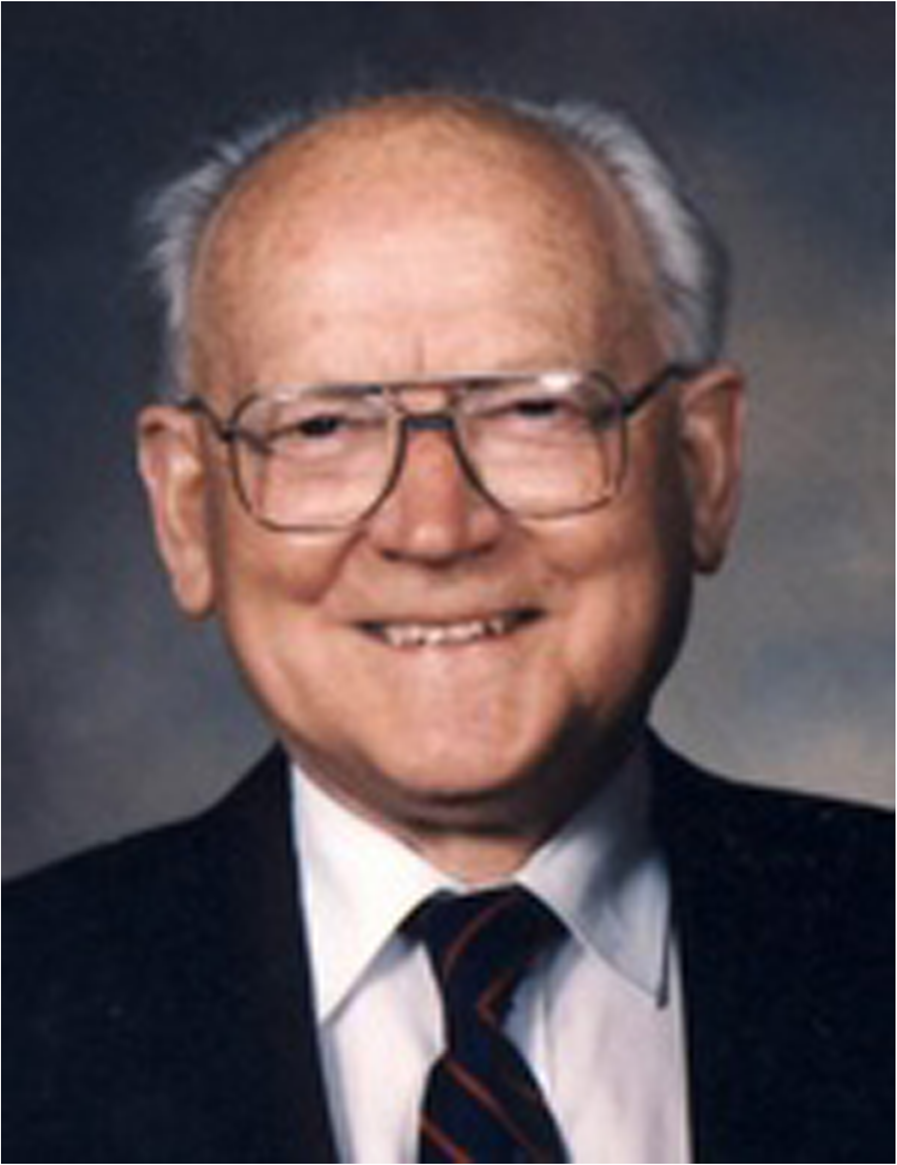
“Stan “Hughes

**Fig. 17:**
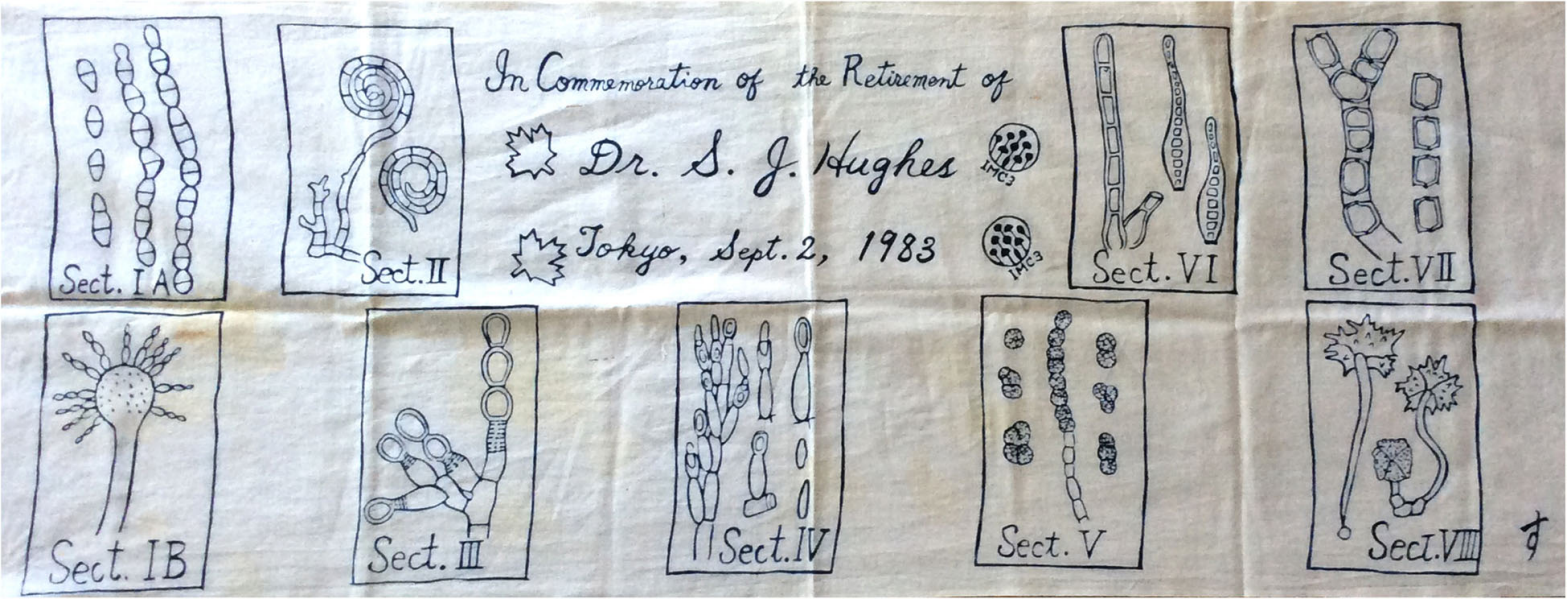
Banner designed to commemorate “Stan” Hughes retirement showing the categories of conidiogenesis he recognized and presented to him at IMC2

Stan received numerous honours and awards, amongst which are the Jakob Eriksson Gold Medal of the Swedish Academy of Science (1969) and the George Lawson Medal of the Canadian Botanical Association (1981). He was a Vice-President of the IMA from its foundation in 1971 to 1983. Since his retirement in 1983, he continued as an honorary research associate, at the now Biosystematics Research Centre of Central Experimental Farm, and in 1986 he was elected a foreign member of the Linnean Society of London. Tributes were paid to him in *IMA Fungus* on the occasion of his appointment to the Order of Canada (1(2): (17), 2010) and his centenary (9(2): (64)–(65), 2018). His library was presented to the National Botanic Garden of Wales in 2009, at a ceremony he attended and where it is kept as a special collection.

He last met many of his long-standing mycological colleagues at Keith Seifert’s retirement party in Ottawa on 30 October 2019, just a week before he died, where he was also able to chat with David Malloch, Kris Pirozynski, and Scott Redhead amongst others – and where Rob Samson was a surprise guest. Always very much a Welshman, and active in the Welsh community in Ottawa, he enjoyed having an address in his honour given in Welsh by E. B. Gareth Jones in Tokyo during IMC3 on 2 September 1983 to celebrate his retirement; in response, he commented how fortunate he had been to be paid all his life for pursuing his hobby.

“Stan” was truly one of the “Greats” in mycology, and his excited and insightful enthusiasm will be missed and, very much a family man and loving husband, our thoughts are with his wife of 61 years Lyndell and his children and grandchildren at this time. On learning of his passing, Gareth W. Griffith suggested a fitting epitaph might be “Lle bu hwn, mae byd gwell” [where this man went, the world was better].

[*A personal reflection on Stan and his life by one of his closest mycological colleagues for many years, Kris Pirozynski, is included under Correspondence in this edition of* MycoNews*.]*

## Jos Wessels (1934–2019)

(Fig. 18)

Jos Wessels passed away on 30 October 2019 at the age of 85 years. Jos studied biology in the 1950s in Leiden. As part of these studies he did an internship with Albert J. Kluyver in Delft, linking him directly to the ‘Delft School of Microbiology’. During this internship he studied oxidative phosphorylation in *Schizophyllum commune*, resulting in his first publication in 1959. He continued working with this mushroom-forming fungus until his retirement in 1999. In 1965 Jos defended his PhD thesis at Leiden University on the morphogenesis and biochemical processes in *Schizophyllum commune,* after which he moved for 1 year to the USA. Here, he worked together with Donald Niederpruem (Indiana University) and Salomon Bartnicki Garcia (University of California at Riverside). Jos and Salomon became friends, accompanied by many years of intense discussions on tip growth and cell wall synthesis in fungi. Jos was appointed full professor at Groningen University in 1970, where, together with Hans Sietsma and Onno de Vries, he established an important centre for fungal research studying hyphal growth and morphogenesis. Hydrophobins were one of their most important discoveries; these proteins have since been widely studied throughout the world, as indicate by 11,000 hits in Google Scholar.

**Fig. 18:**
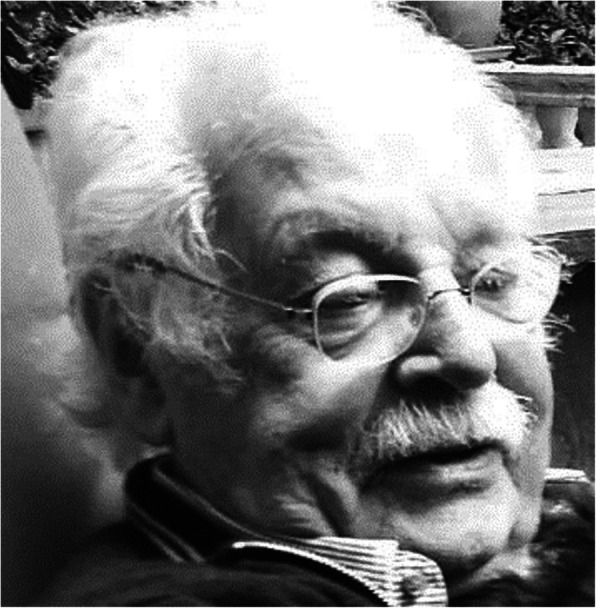
Jos Wessels. Photo: Jan Dijksterhuis

During his career, Jos was head of the Department of Biology and Dean of the Faculty of Sciences of Groningen University. He also served in boards and was active as an editor. He was co-founder of *Experimental Mycology* (now *Fungal Genetics & Biology*) and was part of the scientific committee of the then Centraalbureau voor Schimmelcultures. He was an honorary member of both the British Mycological Society and of the Mycological Society of America.

After his retirement, Jos studied the relation between religion and science with an interest in physics, cosmology, and church fathers, leading to his book *Religion and Science are allies*.

Jos was known as a brilliant teacher, characteristically wearing a lab coat during his lectures. Above all, he was one of the leading scientists in fungal biology; introducing not only the hydophobins, but concepts such as the steady state growth theory and bulk flow secretion of proteins.

**Han A. B. Wösten**

(H.A.B.Wosten@uu.nl)

## BOOK NEWSFungal Biodiversity.

### Edited by Pedro W. Crous, Gerard J. M. Verkley, Johannes Z. Groenewald, and Jos Houbraken. 2019. [Westerdijk laboratory manual series no. 1 (2nd edn).] Utrecht: Westerdijk fungal biodiversity institute. Pp. 425, illustr. (most in colour). ISBN 978–94–91,751-16-5. Price: 75 € (Fig. 19)

When the first edition of this manual appeared in 2009, it was greeted as “a boon to all involved in running practical mycology classes (*IMA Fungus* 1(1): 15, 2010). Now, 10 years on, approaching twice the number of pages, and no longer spiral-bound, that is still the case today.

**Fig. 19:**
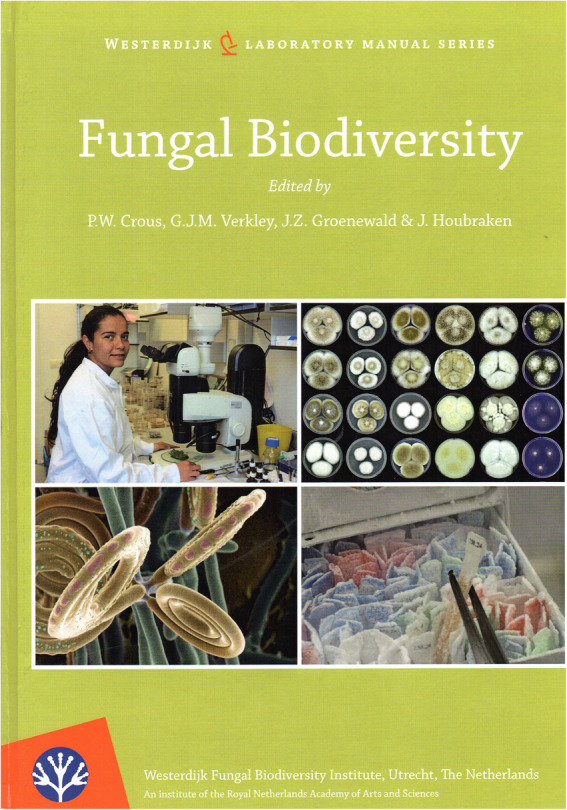
*Fungal Biodiversity* (2019)

Following the successful format of the first edition, the manual starts with a brief overview of species and generic concepts, and then proceeds to a superbly illustrated overview of kingdom *Fungi*, and organisms treated as fungi (again sadly omitting slime moulds), down to order, family, and in some cases genera of particular applied mycological importance. The coloured illustrations of life-cycles are especially finely executed, and I can see them being utilized by many teachers.

A series of chapters on methods follows, ranging from basic aseptic working, microscopic methods, culturing, staining, illustrating, and induction of sporulation to the various molecular phylogenetic approaches; still perhaps the most succinct hands-on guide to how to proceed for those starting to use DNA and other molecular characters in fungi. For a future edition it would be useful to have an equivalent treatment of microchemical techniques for the determination of extrolites. Another chapter that could have been expanded and updated is that on nomenclature which is allotted a mere 3.5 pages; this was unfortunately prepared before the Shenzhen Code appeared last year, and does not take account of changes made in that and also at the subsequent International Mycological Congress in 2018 -- including the wide protection that can be given to lists of name to improve stability, mandatory registration of new typifications, the new governance arrangements, and the discontinuation of the use of the “: “to indicate the sanctioned status of a name.

A chapter on ecological groups of fungi describes the habitats and methods of studying fungi in them, especially isolation into pure cultures, and with a focus on microfungi. It would have been valuable to include methods for some other ecological groups, such as mycorrhizal fungi on roots, lichen-forming fungi on rocks, and foliicolous fungi on leaf surfaces. Fungi of importance in food spoilage and of medical importance are the two key aspects featured in a chapter on fungi of applied importance. Here, I found the treatment of fungi in the air and indoors especially valuable in covering a wide spectrum of sampling methods.

Fifty-five pages are devoted to a glossary (including illustrations of terms for spore shapes and colony characteristics), mycological media, and especially references which cover 40 pages in smaller type. A superbly illustrated section, occupying almost a quarter of the book, treats “reference taxa”, with a double-page spread devoted to 69 species drawn from diverse fungal groups, primarily microfungi that grow in culture and belong to economically important genera, with information on their cultivation, characters, distributions, and ecology, along with key references. A comprehensive index concludes the work.

In order to make the work as authoritative as possible, the editors have involved no less than 24 leading mycologists from around the world as collaborators. This manual is even more of a boon to those teaching practical mycology classes than the 2009 edition, especially those with an emphasis on microfungi that grow in culture. It would be wonderful if the next edition could be allowed to swell to embrace an even greater range of fungi!

## Fungi of Temperate Europe.

### By Thomas Læssøe and Jens H. Petersen. 2019. Princeton, NJ: Princeton University press. 2 vols. Pp. 1715, illustr (col.). ISBN 9878–0–692-28,037-3 [two-volume set]. Price: US $ 110, £ 95 (Fig. 20)

The back cover states that “*Fungi of Temperate Europe* is one of the most comprehensive mycological guides ever published”. This is undeniably so, and it is also innovative in its approach to identification setting new standards for field guides. This is perhaps not so surprising when it combines the expertise of Thomas Læssøe, one of the most experienced field mycologists in Europe, and that of Jens Petersen, not only an exceptional mycologist but an outstanding photographer whose lavish 2012 *The Kingdom of Fungi* was greeted with huge acclaim (see *IMA Fungus* 4(1): (26), 2013).

**Fig. 20:**
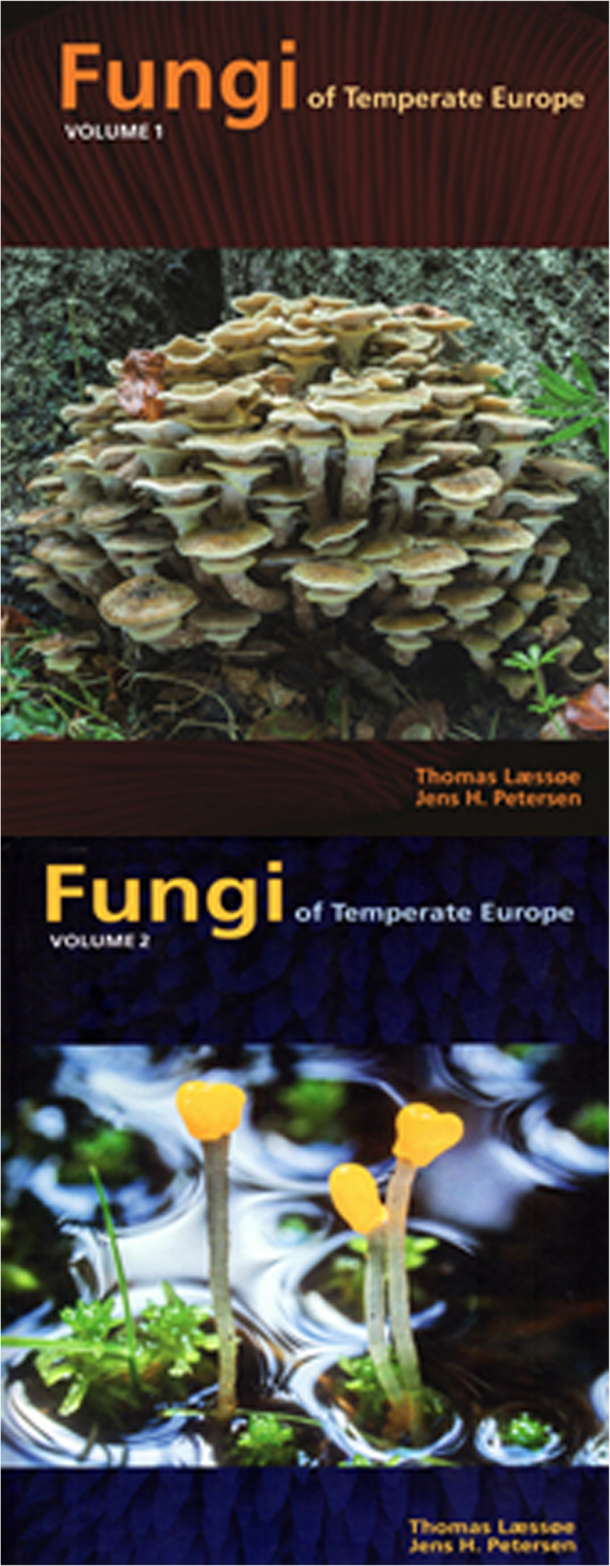
Fungi of Temperate Europe *(2019)*

Over 2800 species are illustrated and treated in detail, and around another 1500 are discussed in the text as potential look-alikes. Most attention is devoted to macromycetes, including discomycetes, but there are also selections of pyrenomycetes, laboulbeniomycetes, plant pathogens, moulds, slime-moulds, and lichen fungi to give a broad view of the range organisms mycologists study. The over 7000 full-colour photographs, all taken by the authors or their colleagues, are of fresh collections taken in the field, generally presented at 2–3 per page, and can only be described as stunning; many are amongst the best yet published for a significant proportion of the species included. Notes on the macro- and microscopic features are provided for each species, together with information on their ecology and distribution in the region. A series of five symbols is placed at the start of species entries to indicate degrees of edibility or poisonousness. I was especially pleased to see that no author citations were included after the scientific names; these are quite superfluous in the modern age as full bibliographic information if needed is freely available through the *Index Fungorum* database (www.indexfungorum.org/). Common names understandably not provided as there are so many in the different European languages.

The authors have collaborated for many years in the production of an innovative downloadable computer-based identification system, *MycoKey* (http://www.mycokey.com/), which has gone through various editions and now includes many more species than treated in this book and keys to some 1000 genera. They have put this experience to great effect in how the present volumes are arranged and how they can facilitate identification. Eighty “form groups” are recognized, each of which starts with an innovative wheel with notes on the whole group in the centre, a radially divided circle with sketches of diagnostic microscopic features such as cystidia and spores, and at the circumference further notes on taxa within that radius with close-ups of key features and photographs of exemplar species. I tried these out with some specimens I collected locally, and was impressed how well they worked. When the right group is reached, however, it is then a matter of studying the photographs and descriptions to make an identification. The series of wheels constitutes an incredibly novel and effective approach to macrofungal identification, combining both macro- and microscopic features, and it is to be hoped it will be emulated in other field guides. I was particularly pleased to see that the wheels can be downloaded separately free of charge (http://www.mycokey.com/Downloads/FungiOfTemperateEurope_Wheels.pdf), and encourage you to try them even if you do not buy the books.

Although in two volumes, these work as a single book, with continuous pagination. The inside front cover of each includes a list of the genera treated within it (with page numbers) which is marvellous for quickly finding them, while the inside back covers have a glossary of often used terms. References cited on the wheels, and a comprehensive index are to be found at the end of the second volume. I was pleased to see that the index had entries by epithet; that makes them especially useful for finding in which recently segregated genera a familiar species is now to be found.

This is a really practical book that will be a boon to field mycologists, especially in temperate regions. It is not, however, a book for the field, as the two volumes together measure 28.5 × 20 × 8.5 cm and weigh 5.4 kg, but something all field mycologists who see it will want to have on the bench near their microscopes when making identifications. The authors are to be congratulated on this truly remarkable achievement, making their many years of practical experience in macrofungal identification available to mycologists at large.

## Fungipedia: a brief compendium of mushroom lore.

### By Lawrence Millman. 2019. Princeton, NJ: Princeton University press. Pp. xiv + 184, 51 illustr. ISBN 978–0–691-19,472-1. Price: US $ 16.95, £ 9.99 (Fig. 21)

It is always a pleasure to welcome texts that aim to “inspire the reader [the] reader to gaze on all fungi, even the nasty ones, in wonder” (p. 179).

**Fig. 21:**
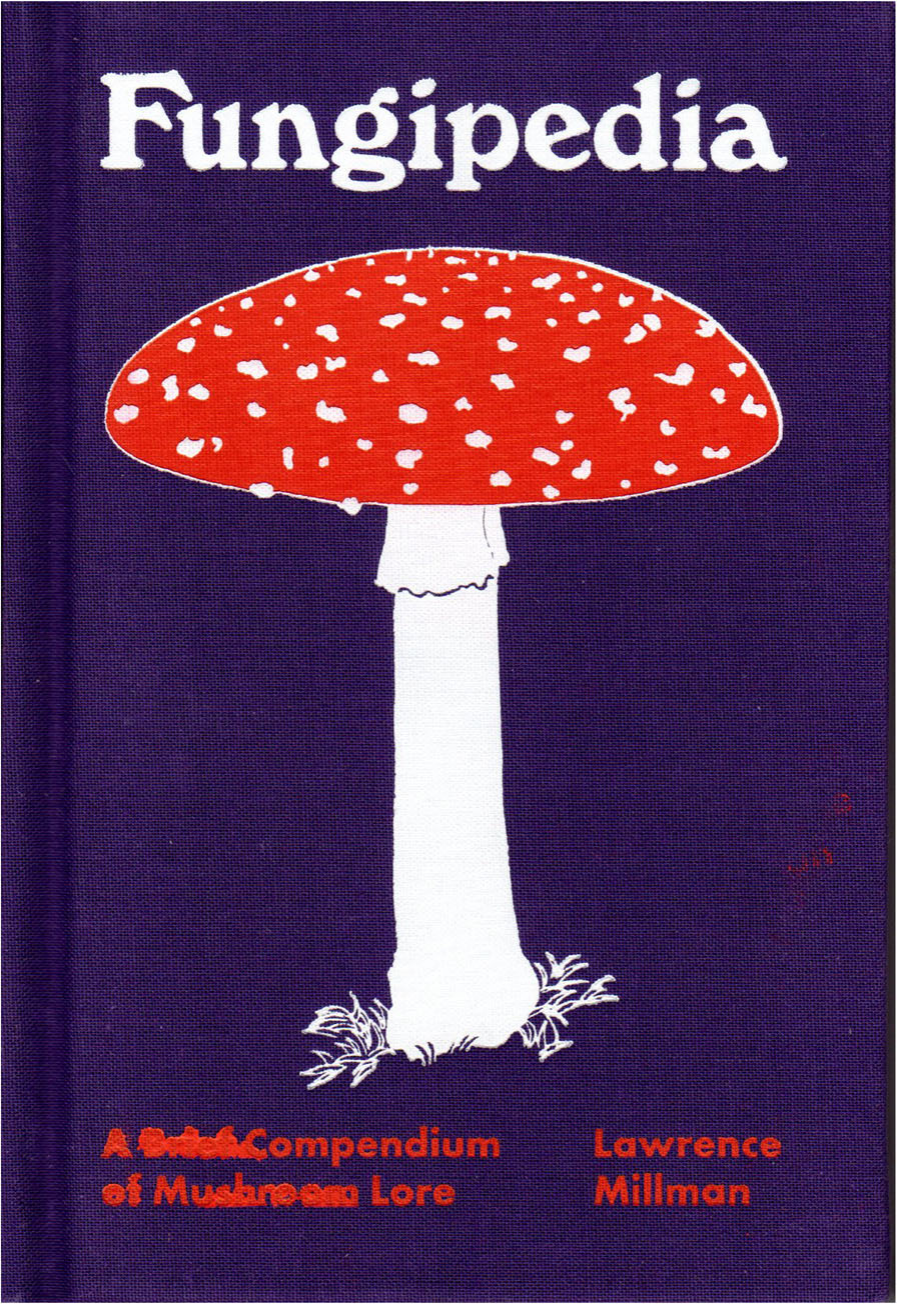
*Fungipedia: a brief compendium of mushroom lore* (2019)

After an engaging and wide-ranging Preface which will surely help to get the reader “hooked” and read on, topics are arranged alphabetically and eclectic, from “agarikon” and “Aksakov” to “zombie ants” and “zygomycetes”. They cover, terms, useful and harmful fungi, particular diseases, folk law, fossils, snippets of ecology and biology, and notes on particular mycologists ranging from the classic to some still active today. There is something of a North American bias in the personages and examples of particular plant diseases, and in general the information is accurate, though there are inevitably some that could have benefitted from up-dating. Entries are mainly about a single page in length, making the book easy to dip into and out of, and broken up by line drawings by Amy Jean Porter. For those that get “hooked” and thirst for more, there is a list of pertinent web-sites and an evidently thoughtfully selected list of “useful references” (though the edition of the *Dictionary of the Fungi* cited is surprising that from 1971).

Lawrence Millman is an experienced author and “amateur” American mycologist, who has evidently travelled and read widely, and also benefitted from contacts with many of today’s leading North American mycologists. From the entry for “amateur”, however, I suspect he would prefer to be referred to just as a “mycophile”, and notes that “amateurs” can often identify fungi much better than professionals devoted to molecular approaches.

In summary, I found this a delightful little book, a mini-encyclopaedia, hardback, and at just 4.5 × 6.75 ins, easy to slip into a pocket and dip in and out of on a bus or metro journey. An ideal Christmas stocking-filler for both mycologists, whether “amateur” or “professional”, and inquisitive naturalists.

## Advances in macrofungi: diversity, ecology and biotechnology.

### Edited by Kandikere R. Sridhar, and Sunil K. Deshmukh. 2019. Boca Raton, LA: CRC press. Pp. viii + 366, illustr. 77 (many colour). ISBN 978–1–138-58,727-4 (hbk), 978–0–429-50,407-5 (ebk). Price: US $ 151.96 (hbk), 40.57 (ebk) (Fig. 22)

Indian mycologists are to be congratulated on the succession of edited texts produced in recent years. Whereas hard-copy books, with a few notable exceptions, are not the sources of choice for mycologists in the most developed countries, they have a particular value in regions where access to electronic services is limited or non-existent.

**Fig. 22:**
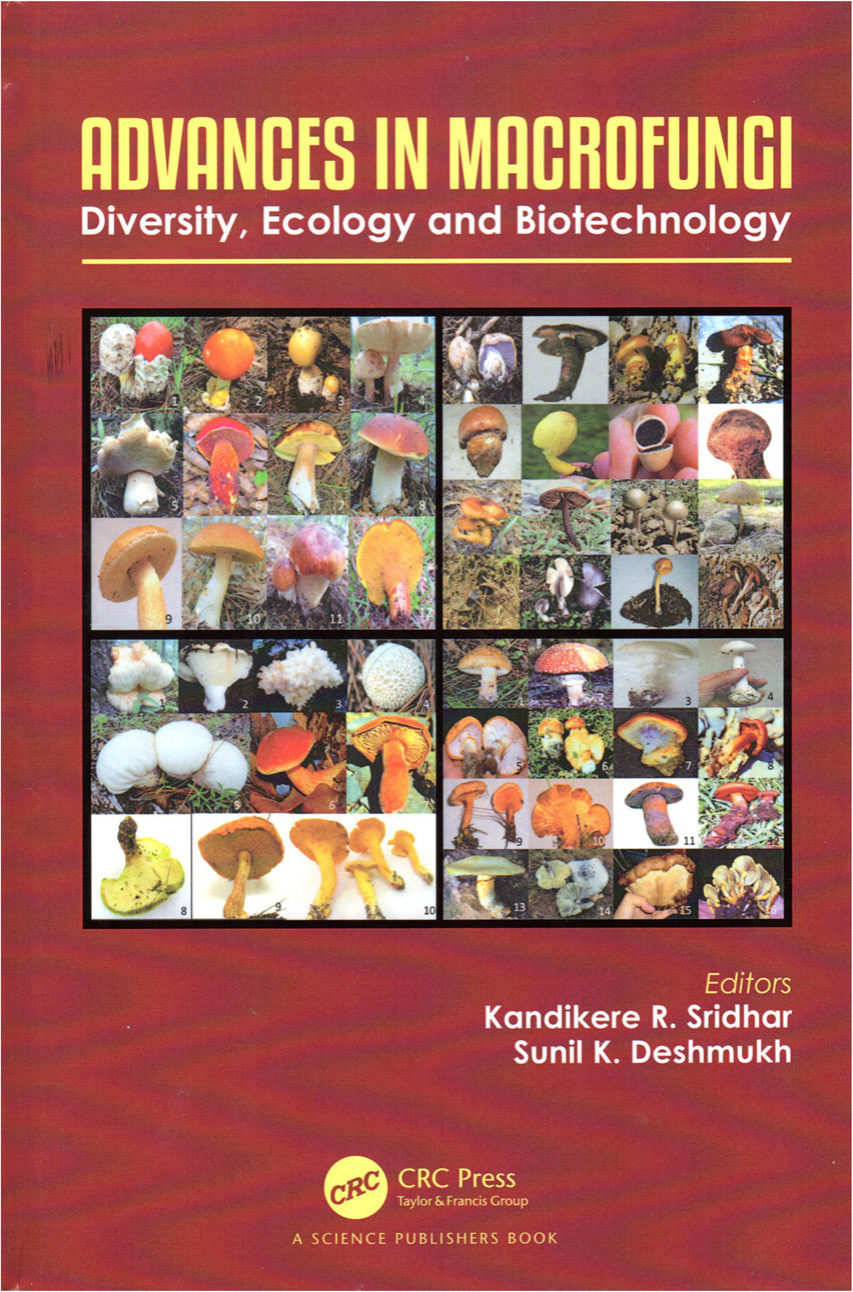
*Advances in Macrofungi: diversity, ecology and biotechnology* (2019)

This is volume has 16 contributions on various aspects of the exploitation and importance of macrofungi involving authors not only from India, but also from Armenia, Ghana, Hungary, Italy, Malaysia, Mexico, New Zealand, the UK, and the USA; a truly international cast. The topics range from the generic to the particular.

The wider-ranging chapters address: the extent of macrofungal diversity (220,000 to 380,000 species estimated); boreal ectomycorrhizal pine forests; roles in terrestrial ecosystems; edible, toxic and medicinal mushrooms from temperate Mexico; exploitation for food, pharmaceuticals, and cosmeceuticals (notably anti-ageing); bioactive metabolites (including extensive lists of polysaccharides, proteins and peptides, and terpenes and their sources); DNA barcoding for molecular identification; nano particle biosynthesis; and proteomics of edible and medicinal mushrooms.

More specific topics covered are: uncertainties in *Ganoderma* taxonomy; interactions of *Auriculoscypha* with insects and plants; nutritional attributes of a wild *Lentinus* and a wild *Termitomyces* species; domestication of *Lentinus squarrosus*; terpenoids from *Russula*; bioactive metabolites from *Phellinus* (with a listing of 86 so far known); and the commercial inoculation of *Pseudotsuga* with ectomycorrhizal-forming *Rhizopogon parksii* and *R. roseolus*,

I found the mix of chapters came together to form a fascinating snap-shot of where we are in utilising macrofungi and insights into aspects that I knew little of before. The general presentation and quality of editing and production is excellent, especially of the colour photographs which often let such works down. Some of the half-tones, however, are unfortunately reproduced rather too small, and numerous author citations after scientific names in some are unnecessarily detracting clutter and would have been better omitted (except in taxonomic contributions). It deserves to find its way into mycological and biotechnological libraries around the world, where it can be available for browsing and will increase awareness of the potential and value of the macrofungal resource.

## MiSAC Matters: 50th anniversary articles.

### Edited by Margaret Whalley and Rachel Exley. 2019. Microbiology in schools advisory committee. Pp. 94, illustr. (colour). [http://www.misac.org.uk/anniversary-articles.html.] Price: free (Fig. 23)

Authoritative reviews on aspects of fungal biology and other areas of and microbiology are not easy to come by, especially free of any charge. The Microbiology in Schools Advisory Committee, which comprises representatives of different educational and learned societies, promotes the teaching of mycology and bacteriology in schools, not only in the UK but also around the world (especially in Asia), by producing materials and advice, and particularly running annual competitions. In order to celebrate 50 years of their activities, the Committee invited a range of leading specialists to contribute a series of 33 topical reviews, no less than 16 of which are devoted wholly or partly to fungi. Amongst the topics treated are: biorecovery of metals, fungi in shopping baskets, emerging diseases, natural products, tree and other plant pathogens, plastic-eating fungi, *Candida*, dung fungi, climate change, lessons from Beatrix Potter, mycoprotein, lichens, and arbuscular mycorrhizal fungi.

**Fig. 23:**
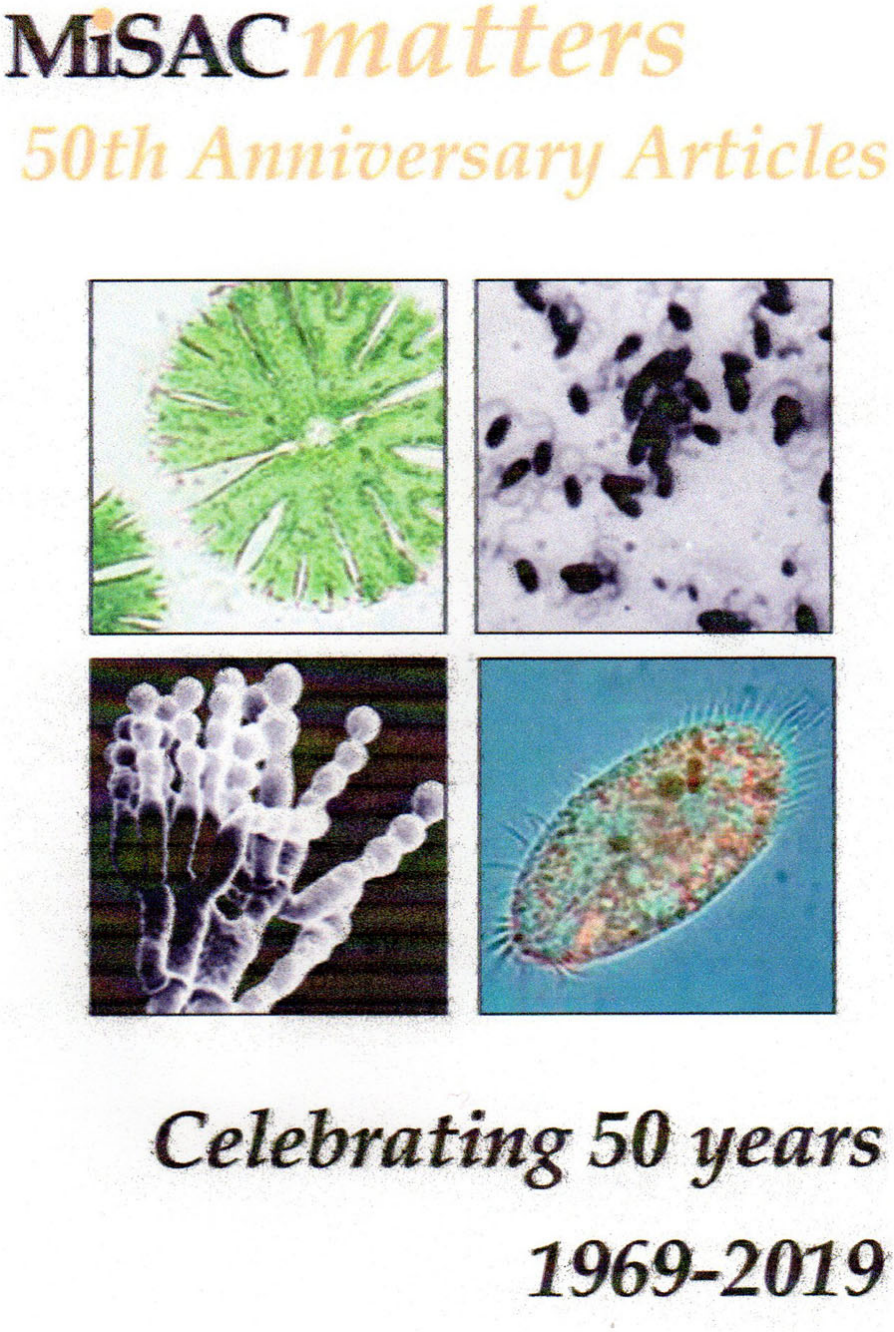
*MiSAC Matter:s celebrating 50 years 1969-2019* (2019)

Articles are written with a senior school audience in mind, but also be a boon to biology teachers working to make their lessons captivating, and well-illustrated in full colour by photographs and/or diagrams. At just 2–4 pages each, they are ideal as quick-reads to download onto an i-phone or tablet. In a Foreword to this collection, Nobel Prizewinner Sir Paul Nurse, surely the most honoured mycologist of the day, applauds “MiSAC’s work to provide a sound, basic foundation to the science and its efforts to encourage awareness and interest in all things microbiological amongst school students.” Long may MiSAC’s much-needed work continue.

## CORRESPONDENCE A personal tribute to Stanley J. Hughes (Llanelli 1918 - Ottawa 2019)

(Fig. 24)

One good thing about a long life is that friends can enjoy your company longer. I was lucky to have had Stan as a friend for more than half a century. But his influence on my life goes back further: to the middle of the last century. Yet the period I remember best is now, a few short moments ago. I sat in their living room, Stan and Lyndell’s, and we chatted. He was a little hard of hearing, a little hard of seeing, but not at all hard of thinking. We chatted about many things, hyphomycetes, sooty moulds, New Zealand, and about life in general. He often stressed what a wonderful life he had. One recurrent theme of our conversations was collecting. Stan was an inveterate collector (hoarder, according to Lyndell) of everything: stamps, coins, bottle tops and his latest consuming passion, the pins. All were neatly classified into groupings reminiscent of the 1953 scheme. But what he missed the most was collecting fungi. He bemoaned the apparent demise of collecting as a prerequisite of any biological study. Stan kept his treasures in a basement room completely clogged with books, albums, boxes, magnifying glasses, etc. A set of stairs led to this sanctum, stairs that Stan climbed several times a day. They kept him mobile, “steady on my pins” he used to say, to the very end. Not a walker or cane in sight.

**Fig. 24:**
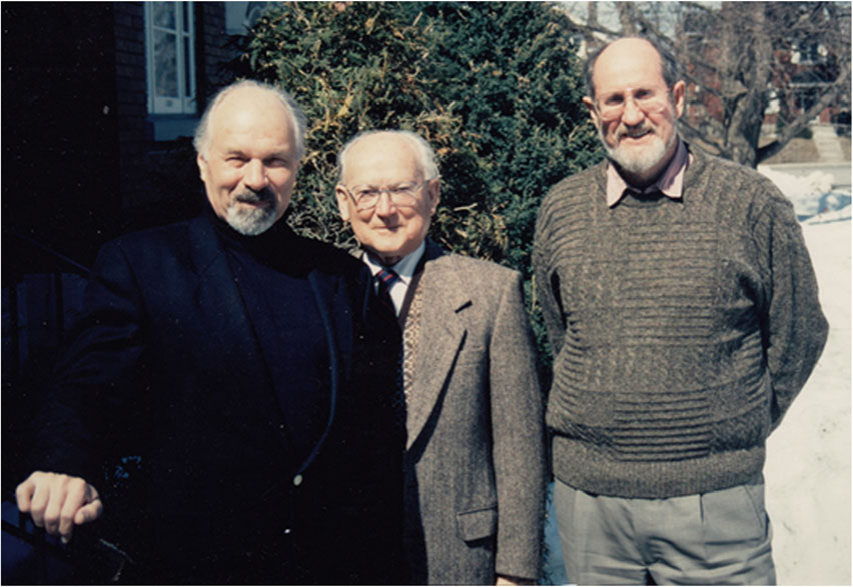
“Stan” Hughes (*centre*) with Kris Pirozynski (*left*) and Charles Stirton, the first Director of the National Botanic Garden of Wales, now the home of the Stanley Hughes Library (*right*); in front of Hughes’ residence in Ottawa, winter 1999

After Keith Seifert’s retirement party on 30 October 2019, which both Stan and Lyndell attended, David Malloch and I drove them home. We exchanged “see you’s” and waved “goodbyes”. A few days later, on 7 November, Stan was gone. I felt as if he had just walked out of the living room, and shut the door on an era.

I am not going to remind you of Stan’s accomplishments and honours. Every mycologist knows it. Instead, I would like to reminisce about Stan’s impact on my own life. I think that all of us mycologists, young and old, will find a bit of Stan in ourselves. My mind is a little dimmed now and my facts are sometimes “alternative”, so I reproduce below a letter I wrote to Stan back in the last century:

5 September 1983

*Dear Stan,*

*This occasion, commemorating your first thirty-one years of service to mycology in Canada, not only gives me the opportunity to join other well-wishers in acknowledging your many outstanding contributions, but also to reflect on my own mycological career which so closely traced yours. Through a series of coincidences we shared interests, colleagues and roofs, years before our first meeting in Ottawa. Like you I developed an interest in hyphomycetes, but so did others. But unlike others, my interest in ‘hyphos’ - like yours - blossomed at the CMI and - like yours- matured in Ottawa.*

*Coincidences? There are a few things that you do not know and the significance of which I did not fully appreciate till now. So, let me take you back in time to Bristol - you know that little place across the river from Llanelli. The year is 1958 and I am well on the way to becoming a bad plant pathologist. One sunny afternoon, which I so vividly remember, T.E.T. Bond points to a drawing of* Ceratocladium microspermum *in a paper that seems oddly out of place among club-rooted turnips and sieved out nematode cysts. “What endless variety of form” he mutters and leaves. The paper has blue covers and an author named Hughes. “Studies on Microfungi. XIII.” Part 13? This, I suppose, explains the “endless variety”. But I am disturbed. I thought I knew everything there was to know about Fungi Imperfecti. Only last year, just before my final exams, I read the chapter devoted to this group of fungi in the approved university text - both paragraphs!*

*Soon after I find myself at the CMI, and at the start of a mycological career. “Hughes worked here, did you know?” No I did not, but it does not take long to discover that Hughes was here. Cabinets overflow with packets from the Gold Coast and Ranmore Common annotated “SJH”. A voice shatters the silence: “I think Hughes said they were annelides”. I am sitting at the far end of a long work bench, last in a row of mycologists peering down monocular tubes of shining brass microscopes. Mine has 1908 stamped at the base and keeps sliding out of focus. I wander if it was his? At the other end of the bench is a door, partly ajar and emitting dense fumes. “Who is there?” “Shhh …its Mr Mason”.*

*I was instructed this morning that I am to work on things called ascomycetes, and spend the day wrestling with the identification of a dry, brownish crumb. Mason comes along and asks what I think of Clements and Shear. I explain that this is my first day, and that I have not yet had the chance to meet the staff. The crumb eventually keys out (in Clements and Shear) as* Bulgaria*.*

*Three years later my first papers come out. They are not on* Bulgaria*, not even on ascomycetes. One is on* Gyrothrix *and, as I write this letter 20 years later, I am astonished to discover how similar* Gyrothrix *is to* Ceratocladium microspermum*. The second paper is on* Beltrania *- the other subject of your “Studies on Microfungi. XIII.”*

*It is 1966, and an unexpected letter arrives from Canada. I am surprised that it is from you because we have not exchanged correspondence before. You ask if I ever considered coming to Canada as a PDF. I reply that I have not. Besides, I do not have a PhD. The exchange concluded, copies are filed to be forgotten. But within six months I am registered for a Ph.D. and within a year I turn up in Ottawa - to work on ascomycetes.*

*You know the rest except, perhaps, that working alongside you, I learned that innovation is a function of intuitive curiosity, patience and analytical thinking. How many times have I witnessed your elation with an idea, watched in awe the ensuing periods of concentration, to be astonished by the elegant simplicity of the solutions.*

*Thank you Stan for the continued inspiration since that day in Bristol twenty-five years ago and, during the last sixteen, for sharing with me your insights, experience, enthusiasm and occasional frustrations which you always tempered with characteristic wit and sense of humour.*

*As ever, Kris*

Stan instilled passions and influenced careers of many. Some do not fully appreciate by how much. I did not. Today, I join his friends and colleagues, and all those whose life he touched, in saying thank you. Goodbye my friend. Happy collecting wherever you are.

**Kris A. Pirozynski**

(jpiroz2@rogers.com)

[*See also the entry for “Stan” Hughes in the “*In Memoriam*” section of this instalment of* MycoNews *above.*]
